# Dynamical Modeling of the Moth Pheromone-Sensitive Olfactory Receptor Neuron within Its Sensillar Environment

**DOI:** 10.1371/journal.pone.0017422

**Published:** 2011-03-02

**Authors:** Yuqiao Gu, Jean-Pierre Rospars

**Affiliations:** INRA, UMR 1272, Physiologie de l'Insecte: Signalisation et Communication, Versailles, France; Center for Genomic Regulation, Spain

## Abstract

In insects, olfactory receptor neurons (ORNs), surrounded with auxiliary cells and protected by a cuticular wall, form small discrete sensory organs – the sensilla. The moth pheromone-sensitive sensillum is a well studied example of hair-like sensillum that is favorable to both experimental and modeling investigations. The model presented takes into account both the molecular processes of ORNs, i.e. the biochemical reactions and ionic currents giving rise to the receptor potential, and the cellular organization and compartmentalization of the organ represented by an electrical circuit. The number of isopotential compartments needed to describe the long dendrite bearing pheromone receptors was determined. The transduction parameters that must be modified when the number of compartments is increased were identified. This model reproduces the amplitude and time course of the experimentally recorded receptor potential. A first complete version of the model was analyzed in response to pheromone pulses of various strengths. It provided a quantitative description of the spatial and temporal evolution of the pheromone-dependent conductances, currents and potentials along the outer dendrite and served to determine the contribution of the various steps in the cascade to its global sensitivity. A second simplified version of the model, utilizing a single depolarizing conductance and leak conductances for repolarizing the ORN, was derived from the first version. It served to analyze the effects on the sensory properties of varying the electrical parameters and the size of the main sensillum parts. The consequences of the results obtained on the still uncertain mechanisms of olfactory transduction in moth ORNs – involvement or not of G-proteins, role of chloride and potassium currents – are discussed as well as the optimality of the sensillum organization, the dependence of biochemical parameters on the neuron spatial extension and the respective contributions of the biochemical and electrical parameters to the overall neuron response.

## Introduction

Olfactory receptor neurons (ORNs) are specialized cells which detect and code for the presence, nature, concentration and temporal fluctuations of volatile molecules in their environment. They convert the odor signal in a sequence of biochemical and electrical events – a process called transduction whose final output is a train of action potentials sent to the brain along the ORN axon. ORNs provide to the brain abundant and subtle information because they detect a large number of odorants with vastly diverse molecular structures and discriminate them based on tiny chemical differences [1]. As a result, the animals can orient towards odor sources or away from them, locate food and preys or avoid predators, and communicate with other members of their species or with other species. Natural odors are usually mixtures of many odorants which are classically divided in pheromones and allelochemicals. Pheromones are emitted and received by individuals of the same species [2], [3] a typical example being sexual pheromones, like that emitted by female moths to attract conspecific males [4], [5], [6]. Allelochemicals are emitted by a species and received by another one, a typical example being flowers whose scents attract pollinators. In both insects and vertebrates, different ORNs transduce pheromones and allelochemicals [7], [8].

**Figure 1:**
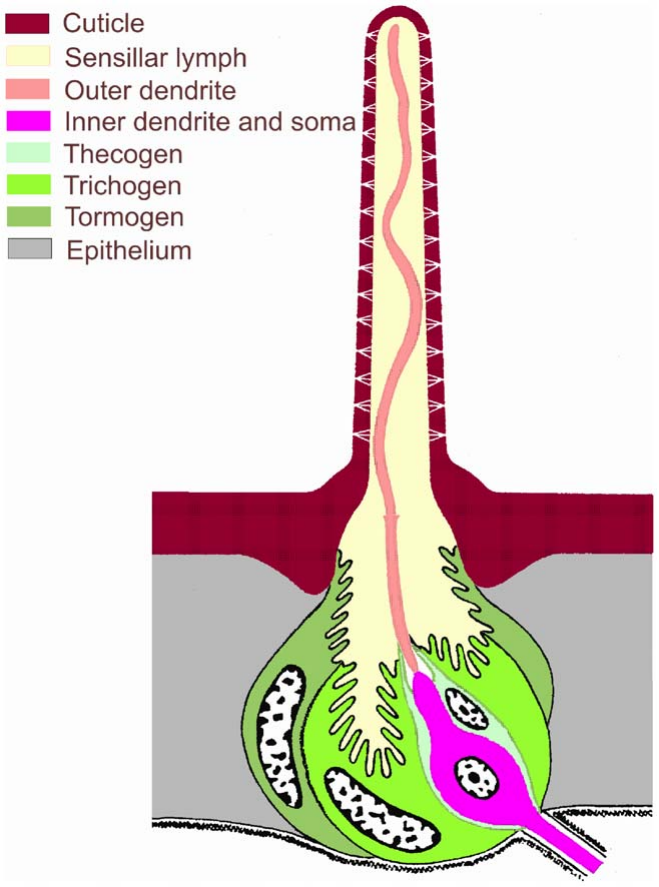
Moth pheromone-sensitive sensillum trichodeum. The sensillum is typically composed of two ORNs and three auxiliary cells (thecogen Th, trichogen Tr and tormogen To). The tight junctions between cells separate the ORN extracellular environment in two parts with different ionic compositions, the sensillar lymph bathing the outer dendritic segment (housing the transduction machinery) and the hemolymph bathing the inner dendrite and soma. Pheromone molecules enter the sensillar lymph through pores in the hair shaft. The sensillar potential SP (ORN electrical response to pheromone stimulation) is recorded between the active electrode, slipped on the cut hair tip in contact with the sensillar lymph, and the indifferent electrode in contact with the hemolymph. Fig. 2 gives an overview of the global electrical organization of the sensillum. Fig. 3 shows part of ORN membrane processes at the molecular level. Modified from [66].

In the present work we study sex-pheromone transduction in moth ORNs. Usually a moth sex pheromone is a blend in a specific ratio of two to three hydrocarbon molecules with 12 to 16 carbon atoms. Several thousand ORNs of the male moth antenna are specialized in the detection of these pheromone components. ORNs are typically associated in pairs and surrounded with accessory cells within specialized hair-like organs called sensilla [9], [10]. ORN electrical responses can be recorded *in vivo* with an electrode slipped on the cut hair tip [11] (Fig. 1); it consists of the so-called sensillar potential (SP) with the superimposed action potentials [12]. Investigating SP generation is essential for a proper understanding of transduction in pheromone and other olfactory sensilla. SP properties depend on a variety of molecular, cellular (ORN level) and multicellular (sensillum level) mechanisms. These mechanisms have been the subject of intensive investigations.

Whatever the odorants and the animal group, the central event of olfactory transduction is the interaction of odorant molecules with membrane proteins − the olfactory receptors (ORs, [13]. Pheromone ORs have been found also in moths [14], [15]. Most ORNs express a single OR gene. Located on the cilia (in vertebrates) and outer dendritic segments (in insects), ORs form the interface between external reactions taking place in the aqueous environment of ORNs (mucus of vertebrates and sensillum lymph of insects, both in contact with the air) and intracellular reactions involving various membrane proteins, cytoplasmic modulators and ions whose main function is to amplify the weak initial signal provided by odorant binding to ORs.

In the main olfactory epithelium of vertebrates, the activated OR bind to a G-protein which activates an effector enzyme (adenylyl cyclase) catalyzing the conversion of ATP to cyclic AMP (cAMP), then cAMP gates a cationic channel permeable to calcium. The increased concentration of Ca^2+^ gates a second depolarizing current borne by chloride ions [16], [17]. In insects the cationic and chloride channels have also been found but they may significantly differ from their vertebrate analogs (for more complete references, see [18], [19], [20], [21]). First, in moths, a G-protein activates an effector (PLCβ [22], [23]) producing two second messengers (IP_3_ and DAG) that open a Ca^2+^ channel and a cationic channel respectively [24], [25], [26], [27], [28], [29], [30], [31], [32]. However, in *Drosophila* it has been shown recently that ORs gate directly a cationic current [33], [34], [35] which casts doubt on the universality of the metabotropic pathway. Three qualitative models of insect transduction have been proposed so far. All combine the metabotropic and ionotropic pathways in very different manners [18], [20], [21]. Second, in moths, a chloride current has also been described [36] although it remains uncertain whether it is depolarizing or repolarizing. Third, other currents are present, for example a large Ca^2+^- and voltage-gated K^+^ current [37], [38], [39] that may contribute to receptor-potential or action-potential generation. The combination of these currents creates the receptor potential (RP) which propagates passively from the ciliary or outer dendritic membrane to the inner dendritic segment, cell body and axon where it triggers action potentials. However, at least in insects, RP generation must take into account the transepithelial potential (TEP) which is generated by the auxiliary cells between the sensillum lymph, which bathes the outer dendrite, and the hemolymph, which bathes the inner dendrite, soma and axon. SP results from the combination of RP and TEP [40].

Moth pheromone sensilla have been the subject of several modeling studies based on extensive experimental data [8], [41]. The available quantitative models describe the diffusion of pheromone molecules in the air [42], the extracellular pheromone transport and degradation in the sensillum lymph [41], [43], [44], [45], [46], [47], the pheromone-receptor interaction ([48], the post-receptor transduction events [18], [49], the action-potential generation [50], [51], the electrical events taking place in the ORN [52], [53]; or in the whole sensillum ([53], [54], including the electrical interaction between ORNs of the same sensillum [55].

These models were based on two lines of enquiry which were developed almost separately. The first line focused on biochemical reactions taking place near and within the outer dendritic membrane, including ionic channels and their modulation, while neglecting the spatial extension of the outer dendrite, i.e. using basically single point models. The second line focused on the electrical phenomena taking place in the sensillum as a whole, considering the geometry of the system but neglecting its biochemical details. In the present work we aimed at connecting these two lines to develop and analyze an integrated model of the sensillum taking into account the molecular mechanisms as described in Gu et al. [18] and the electrical circuits as analyzed in Vermeulen and Rospars [53].

Within this framework several specific questions were addressed. To what extent must the biochemical parameter values estimated from the single-point model be modified when the spatial extension of the outer dendrite is taken into account? What are the relative contributions of the various biochemical components of the cascade and electrical parameters to the response properties of the RP and the SP? In particular, is it true that extracellular events rather than intracellular signaling govern the kinetics of the SP as suggested by Kaissling [41], [45]? What can these models tell us about the still uncertain mechanisms of olfactory transduction? Can the geometry of the moth sensillum be considered optimum?

## Results

The dynamic properties of the moth ORN in its sensillum environment are analyzed in two subsections. In the first subsection a detailed model is presented taking into account the molecular and ionic aspects of transduction. In the second subsection, the complete model is simplified to analyze the effects of the electrical and geometrical parameters.

### 1. Multichannel and multicompartmental sensillum model

#### 1.1. The complete sensillum model

**Figure 2:**
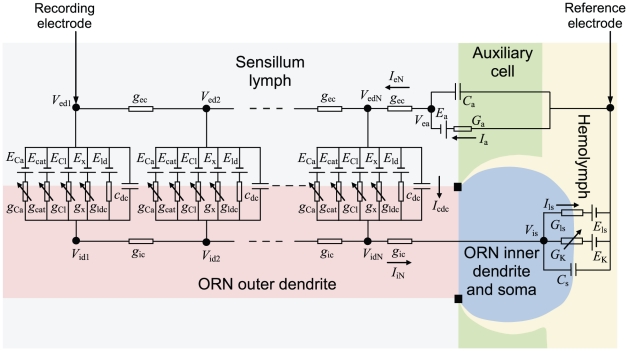
Compartmental model diagram of the moth pheromone-sensitive sensillum. The outer dendrite of the ORN is divided into *N* compartments, the inner dendrite and soma are lumped into a single compartment and the three auxiliary cells are also lumped into one compartment. The equivalent circuit of each outer-dendritic compartment includes the conductances of the external (g_ec_) and internal (*g*_ic_) media and six transmembrane branches for the membrane capacitance (*C*_d_), the leak current and four types of pheromone-dependent currents. Each current is described by a conductance and a constant battery figuring the reversal potential of the permeating ion. The leak current with its constant conductance (*g*_ld_) and battery (*E*_ld_) is responsible for the resting potential. The four pheromone-dependent channels are the IP_3_-gated Ca^2+^ permeable channel (*g*_Ca_, *E*_Ca_), DAG-gated cationic channel (*g*_cat_, *E*_cat_), Ca^2+^-gated chloride channel (*g*_Cl_, *E*_Cl_) and Na^+^/Ca^2+^ exchanger (*g*_x_, *E*_x_). The equivalent circuit of the inner dendrite and soma includes three branches representing the membrane capacitance (*C*_s_), the leak current (*G*_ls_, *E*_ls_) and one pheromone-dependent Ca^2+^-gated K^+^ current (*G*_K_, *E*_K_). The equivalent circuit of the auxiliary cells includes two branches for the membrane capacitance (*C*_a_) and the current (*G*_a_, *E*_a_) responsible for the transepithelial resting potential. The sensillar potential SP is measured between the recording electrode and the reference electrode.

**Figure 3:**
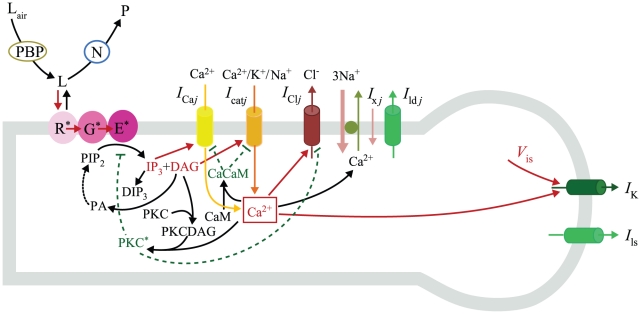
Pheromone transduction cascade. Pheromone molecules in the air (L_air_) are translocated through the sensillum lymph by pheromone binding proteins (PBP) and deactivated (from L to inactive product P) by enzymes (N). These extracellular processes were taken into account in the model as previously described in [45] and [18]. Activation by L of receptors (R*) activates in turn G-proteins (G*) then effector enzymes (E*) that cleave PIP_2_ in IP_3_ and DAG. These second messengers gate Ca^2+^ (*I*_Ca_) and cationic (*I*_cat_) currents. The resulting increase in intracellular Ca^2+^ concentration triggers feedback control of E*, *I*_Ca_ and *I*_cat_ via CaCaM and PKC* and feedforward gating of a Cl^−^ current (*I*_Cl_). Ca^2+^ concentration is regulated by the Na^+^-Ca^2+^ exchanger (NCX). The membrane is depolarized by currents *I*_cat_ and *I*_Cl_ in the ORN outer dendrite (cylindrical compartment on left). It is repolarized by K^+^ (*I*_K_) and leakage (*I*_ld_, *I*_ls_) currents in the inner dendrite and soma (spherical compartment on the right).

The sensillum housing a single ORN is divided in three electrically interconnected parts: (i) the outer dendrite bathing in the sensillum lymph which is the sensory part of the ORN with pheromone transport and degradation, pheromone ORs and ionic channels generating RP, (ii) the non-sensory part of the ORN with the inner dendrite, soma and axon bathing in the hemolymph, (iii) the auxiliary cells separating the outer dendrite and sensillum lymph from the hemolymph. In the present model the auxiliary cells and the relatively short ORN non-sensory part (≈30 µm) are each represented by a single electrical compartment, whereas the long sensory part (220 µm in recording conditions [11]) is modeled by *N* compartments (Fig. 2) instead of a single isopotential compartment in [18]). Each compartment of the outer dendrite includes the complete transduction machinery. As summarized in Fig. 3 it takes into account the translocation of pheromone molecules from air to sensillum lymph, their interaction with the pheromone receptors, their deactivation, the activation of G-proteins and effector enzymes (PLC), the production and degradation of second messengers, the cascade of ionic channels (transient cationic current, a long-lasting chloride current and a delayed potassium current), the feedback inhibition on PLC and channels by protein kinase C (PKC) and Ca^2+^-calmodulin (CaCaM), the central regulatory role of Ca^2+^ and its extrusion. For facilitating comparisons, we kept as starting point the same basic assumptions as in [18], notably the assumptions that the outer-dendritic Cl^−^ current is depolarizing and the inner-dendritic K^+^ current is repolarizing (alternative assumptions are considered in the second subsection). This complete model is described by eqs. (1)–(19) in the Methods section.

The values of geometrical and passive electrical parameter are given in Tables 1 and 2. The values of the electrical and biochemical parameters describing the ionic channels are given in Table 3. Values in Table 3 were fitted to SP experimental measurements performed in *Antheraea polyphemus* [45], [56] knowing their physiologically acceptable ranges. Most of them are the same as in Gu et al. [18]. However, all electrical parameter values determining the strength of the response had to be modified to take into account the subdivision of the outer dendrite in *N* identical compartments (this is the main difference with our previous three-compartmental model). First, the maximal conductances of the ionic channels present in the outer dendrite found for a single-compartment dendrite had to be distributed between *N* compartments. For reasons explained in the second subsection, we chose *N*  =  40; so, maximal conductances in [18] were divided by 40. Similarly, the values of extracellular (*g*_ec_), intracellular (*g*_ic_) and leak conductances (*g*_ldc_) were calculated for forty compartments based on their corresponding global values *G*_i_, *G*_e_ and *G*_ld_ for the whole outer dendrite (based on [48]; see Table 2). Second, to obtain better fits to experimental values of SP, as quantified by the cost function (42) (see Methods section), two other parameters were decreased: the maximal synthesis rate *s*_M_ (connecting the effector enzyme to the cationic channels) and the maximal conductance *G*_MK_ of K^+^ current (in inner dendrite and soma). Third, for fine tuning, other parameters were modified: principally the equilibrium potential of the Na^+^/Ca^2+^ exchanger (*E*_x_) and the maximal conductances of cationic (*G*_Mcat_) and chloride (*G*_MCl_) currents, whereas their Hill coefficients (*n*_cat_ and *n*_Cl_) and the maximum conductance of Ca^2+^ channels (*G*_MCa_) were modified by less than 10%. All other values were kept unchanged with respect to Gu et al. [18].

**Table 1:** Basic geometrical and electrical parameters of the sensillum model.

	Parameter	Symbol	Unit	Value	Reference
Outer dendrite	Length	*L*_d_	µm	220^a^	[11]
	Mean diameter	*D*_i_	µm	0.475^a^	[67]
	Membrane resistivity	*ρ*_ld_	Ωcm^2^	7500	[48]
Media of outer dendrite	Hair mean inside diameter	*D*_e_	µm	1.8^a^	[67]
	Sensillar lymph resistivity	*ρ*_e_	Ωcm	40	[48]
	Intracellular resistivity	*ρ*_i_	Ωcm	40	[48]
Inner dendrite and soma	Lateral area	*S*_s_	µm^2^	144^b^	[68]
	Membrane resistivity	*ρ*_ls_	Ωcm^2^	1000	[48]
	Equilibrium potential of leak currents	*E*_ls_	mV	−62	[38]
Auxiliary cells	Capacitance of apical membrane	*C*_api_	pF	30	[68], [69], [70]
	Resistance of apical membrane	*R*_api_	MΩ	300	[68], [69], [70]
	Resistivity of basolateral membrane	*ρ*_bas_	Ωcm^2^	100	[68], [69], [70]
	Resistance of basolateral membrane	*R*_bas_	MΩ	25	[68], [69], [70]
	Equilibrium potential^c^	*E*_a_	mV	−35	[71]
All membranes	Specific capacitance	*c*	µF/cm^2^	1	[72]

a Morphometric data on ORN and hair are for sensillum trichodeum cell A (with thick dendrite and large action potentials) of *Antheraea polyphemus* in tip-recording conditions, i. e. with cut hair tip.

b Modified from data on *Antheraea pernyi*.

c Gives rise to the transepithelial potential.

**Table 2:** Geometrical and electrical parameters derived from Table 1.

	Parameter	Symbol	Unit	Value	Explanation
Outer dendrite	Lateral area (membrane)	*S*_d_	µm^2^	328	π *L*_d_ *D*_i_
	Volume (intracellular)	*V*_d_	µm^3^	38	π *L*_d_ *D* ^2^_i_/4
	Equilibrium potential of leak currents	*E*_ld_	mV	−97	*E*_ls_ + *E*_a_
	Capacitance per unit length a	*c*_d_	F.cm^−1^	1.49×10^−10^	*c*_m_ π *D*_i_
	Capacitance b	*C*_d_	pF	3.28	*c*_m_ *S*_d_
	Resistance per unit length a	*r*_ld_	MΩ.cm	50.26	*ρ*_ld_/π*D*_i_
	Membrane resistance and leak conductance b	*R*_ld_ *G*_ld_	GΩnS	2.280.4373	*ρ*_ld_/*S*_d_1/*R*_ld_
	Membrane space constant	*λ*	µm	455	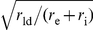
	Membrane time constant	*τ*	ms	7.5	*_r_*_ld_ *c*_d_
	Electrotonic length	*l*_d_	*−*	0.484	*_L_*_d_/*λ*
Media of outer dendrite	Sensillar lymph resistance per unit length a	*r*_e_ *g*_e_	GΩ.cm^−1^nS.cm	1.690.592	4*ρ*_e_/π(*D* ^2^_e_ – *D* ^2^_i_)1/*r*_e_
	Sensillar lymph resistance and conductance^c^	*R*_e_ *G*_e_	MΩnS	37.426.77	*r*_e_ *L*_d_1/*R*_e_
	Intracellular resistance and conductance per unit length a	*r*_i_ *g*_i_	GΩcm^−1^nS.cm	22.570.0443	4*ρ*_i_/π*D* ^2^_i_1/*r*_i_
	Intracellular resistance and conductance c	*R*_i_ *G*_i_	MΩnS	4972.011	*r*_i_ *L*_d_1/*R*_i_
Inner dendrite and soma	Membrane resistance and leak conductance	*R*_ls_ *G*_ls_	MΩnS	6941.44	*ρ*_s_/*S*_s_1/*R*_s_
	Capacitance	*C*_s_	pF	1.44	*c*_s_ *S*_s_
	Dimensionless ratio	*r*_in_	−	0.900	(*R*_ls_ + *R*_a_)/λ (*r*_e_ + *r*_i_)
	Dimensionless ratio	*a*	−	0.3018	*R*_a_/(*R*_ls_ + *R*_a_)
Auxiliary cells	Area of apical membrane	*S*_api_	µm^2^	3000	*C*_api_/*c*
	Resistivity of apical membrane	*ρ*_api_	Ωcm^2^	9000	*S*_api_ *R*_api_
	Area of basolateral membrane	*S*_bas_	µm^2^	400	*ρ*_bas_/*R*_bas_
	Capacitance of basolateral membrane	*C*_bas_	pF	4	*cS*_bas_
	Resistance and conductance	*R*_a_ *G*_a_	MΩnS	3253.1	*R*_api_ + *R*_bas_1/*R*_a_
	Capacitance	*C*_a_	pF	3.53	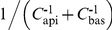

a Notations *c*_d_, *g*_e_, *g*_i_, *g*_ld,_ (and *r*_e_, *r*_i_, *r*_ld_) apply to the “per unit length” (unidimensional cable) description. The corresponding parameters “per compartment” are denoted *c*_dc_, *g*_ec_, *g*_ic_, *g*_ldc_ (and *r*_ec_, *r*_ic_, *r*_ldc_).

b Conductance and capacitance for the whole outer dendrite. For a single compartment they are *c*_dc_  =  *C*_d_/*N*, *g*_ldc_  =  *G*_ld_/*N*, where *N* is the number of compartments in the outer dendrite.

c Resistances and conductances for the whole internal medium of outer dendrite and whole sensillar lymph. For a single compartment the corresponding conductances are *g*_ec_  =  *NG*_e_ and *g*_ic_  =  *NG*_i_.

**Table 3:** Parameters of second messengers and ionic currents.^a^

	Parameter	Symbol	Unit	Value^b^
IP_3_ and DAG	Maximal synthesis rate	*s*_M_	s^−1^	653 (933)
IP_3_-gated Ca^2+^ current *I*_Ca_	Eq. potential Ca^2+^	*E*_Ca_	mV	140
	Maximal conductance	*G*_MCa_	nS	0.137 (0.14)
	EC_50_ for IP_3_	*K*_mCa_	µM	3.48
	Hill coefficient for IP_3_	*n*_Ca_	−	1
	Maximal inhibition	*i*_MCa_	−	3.08
	IC_50_ for CaCaM	*K*_iCa_	µM	0.61
	Hill coef. for CaCaM	*n*_iCa_	−	2.51
DAG-gated cationic current *I*_cat_	Eq. potential cations	*E*_cat_	mV	0
	Maximal conductance	*G*_Mcat_	nS	0.877 (1.23)
	EC_50_ for DAG	*K*_mcat_	µM	0.0104
	Hill coefficient for DAG	*n*_cat_	−	0.776 (0.86)
	Maximal inhibition	*i*_Mcat_	−	53.2
	IC_50_ for CaCaM	*K*_icat_	µM	0.0377
	Hill coef. for CaCaM	*n*_icat_	−	0.818
Ca^2+^-gated Cl^−^ current *I*_Cl_	Eq. potential Cl^−^	*E*_Cl_	mV	−11.5
	Maximal conductance	*G*_MCl_	nS	12.1 (16.8)
	EC_50_ for Ca^2+^	*K*_mCl_	µM	81.2
	Hill coefficient for Ca^2+^	*n*_Cl_	−	1.443 (1.52)
	Maximal inhibition	*i*_MCl_	−	1.4
	IC_50_ for PKC*	*K*_iCl_	µM	0.06
	Hill coef. for PKC*	*n*_iCl_	−	1.1
Ca^2+^ extrusion *I*_x_	Equilibrium potential	*E*_x_	mV	−25.7 (−17.1)
	Maximal conductance	*G*_Mx_	nS	2.21×10^−3^
	EC_50_ for Ca^2+^	*K*_mx_	µM	0.54
	Hill coefficient for Ca^2+^	*n*_x_	−	0.605
Ca^2+^- and voltage-gated K^+^ current *I*_K_	Eq. potential	*E*_K_	mV	−62
	Maximal conductance	*G*_MK_	nS	1.6091 (4.88)
	EC_50_ for K^+^ (inner)	*K*_mK_	µM	2.803×10^−4^
	Coef. of voltage depend.	*A*_K_	mV	12.5
Conversion factors Ca^2+^	Charge to concentration	*F*	µM pC^−1^	136.37
	For IP_3_-gated channels	*f*_Ca_	µM pC^−1^	4.87
	For DAG-gated channels	*f*_cat_	µM pC^−1^	2.50

a All currents are in the outer-dendritic membrane (in contact with the sensillum lymph), except the Ca^2+^- and voltage-gated K^+^ current which is in the inner-dendritic and somatic membranes (in contact with the hemolymph).

b Values are the same as in Gu et al. [18] except for *G*_MCa_, *G*_Mcat_, *n*_cat_, *G*_MCl_, *n*_Cl_, *E*_x_ and *G*_MK_ (then, the previous values in [18] are given in parentheses). The maximal conductances *G*_MCa_, *G*_Mcat_, *G*_MCl_, *G*_Mx_ are given for the whole outer dendrite; for a single compartment the corresponding values are *g*_MCa_  =  *G*_MCa_/*N*, *g*_Mcat_  =  *G*_Mcat_/*N*, *g*_MCl_  =  *G*_MCl_/*N* and *g*_Mx_  =  *G*_Mx_/*N*, where *N* is the number of compartments.

#### 1.2. RP and SP in the complete sensillum model

**Figure 4:**
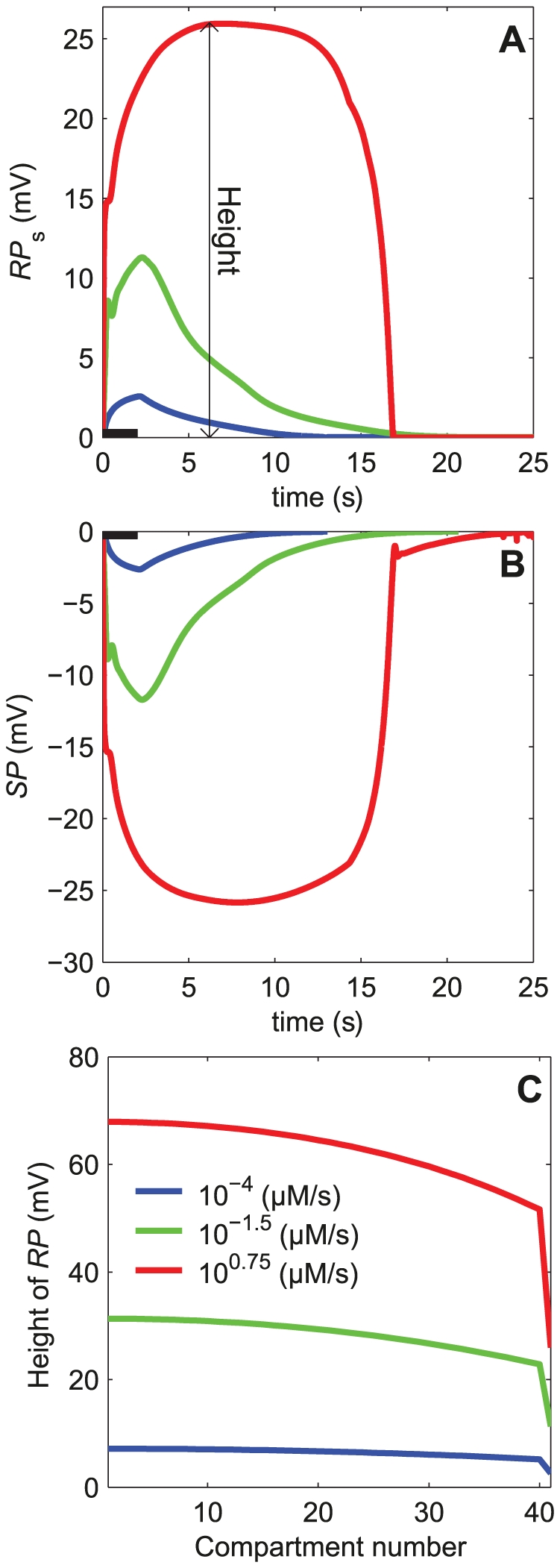
Simulation results of the complete 42-compartment sensillum model shown in Figs. 2and 3. (A) Kinetics of the receptor potential at the ORN soma *RP*_s_. (B) Kinetics of the sensillar potential *SP*_1_ at the tip of the outer dendrite. (C) Height of RP along the ORN (from dendrite tip to soma). Compartment 41 is the inner dendrite and soma. In all plots, kinetics and heights of the potentials are shown at three pheromone uptakes: 10^−4^, 0.032 and 5.6 µM/s. Heights in C are taken at the maximum of the kinetics in A and B. The bars from 0 to 2 second along the time axis in A and B indicate the pheromone stimulation period.

The variation in time of the transmembrane (RP) and transepithelial (SP) potentials were simulated in response to two-second square pheromone pulses of various heights. Because sensilla are flux detectors [43] it is convenient to express the pulse height as a flux or uptake *U* in molarity per second. Simulations for 26 uptakes separated by 0.25 log units from 10^−4.75^ to 10^1.5^ µM/s were run. Three examples of the resulting kinetics are shown in Fig. 4A–B at 10^−4^, 10^−1.5^, and 10^0.75^ µM/s. RP at soma (*RP*_s_, Fig. 4A) and SP (measured at the tip of the outer dendrite, Fig. 4B) present similar kinetics but of opposite signs. Upon stimulation onset, *RP*_s_ (defined by eq. 18 in Methods section) increases rapidly to its maximal value then, after stimulation offset, gradually decreases to zero (Fig. 4A), while *SP* (defined by eq. 19) decreases rapidly to its minimal value then gradually returns to zero (Fig. 4B). The maximum of RP and minimum of SP depend on the pheromone uptake (three uptakes from low to high are shown in Fig. 4); they are about 30 and −30 mV respectively at the highest uptake. These kinetic curves were summarized with three numbers: maximum height (in mV), rising time (τ_rise_) from stimulation onset to half-maximal response and falling time (τ_fall_) from stimulation offset to half-maximal response. Half-maxima were chosen because the times at which RP and SP reach their maxima and return to baseline cannot be determined with precision.

The height (maximum) of RP declines along the outer dendrite, being higher at the tip of the outer dendrite than at its base, then, it declines further at the inner dendrite and soma (Fig. 4C). The latter decline is due to the purely passive nature of the latter segment, which results in an exponential fall of the potential along its length, and to the change of the reference point (extracellular potential), which is in the sensillar lymph for RP along the outer dendrite and in the hemolymph for *RP*_s_.

**Figure 5:**
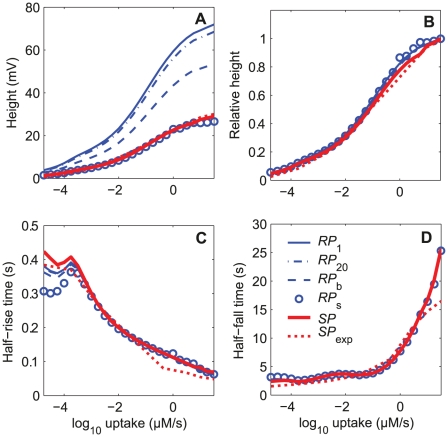
Dose-response characteristics of the receptor potential RP and sensillar potential SP of the complete 42-compartment model. RP and SP in response to 2-s square pulses of pheromone at various uptakes from 10^−4.75^ to 10^1.5^ µM/s. (A) Heights in mV. (B) Relative heights. (C) Half-maximum rising times (*τ*_rise_). (D) Half-maximum falling times (*τ*_fall_). RP is shown at three compartments located at the tip (*RP*_1_), mid-length (*RP*_20_) and base (*RP*_b_) of the outer dendrite, at the inner dendrite and soma (*RP*_s_). Predicted *SP* (−*SP*_1_) is compared to experimentally measured data *SP*_exp_ provided by K.-E. Kaissling ([45], [56]).

**Figure 6:**
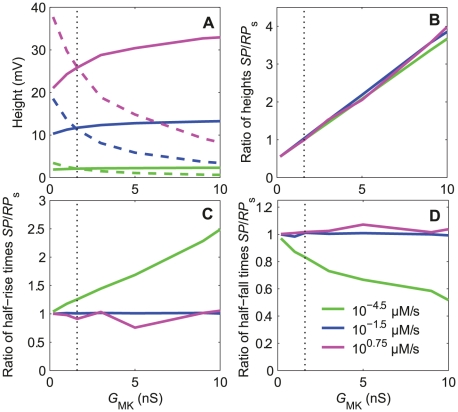
Effects of maximum conductance *G*_MK_ on the response characteristics of RP at soma (*RP*_s_) and SP. The repolarizing conductance *G*_MK_ is the Ca^2+^- and voltage-dependent potassium conductance located at the inner dendrite and soma (the corresponding current *I*_K_ is shown in Fig. 3). (A) The height of *SP* (solid lines) increases, while that of *RP*_s_ (dashed lines) decreases with *G*_MK_ respectively. (B) The ratio of amplitudes SP/*RP*_s_ increases linearly with *G*_MK_ at all uptakes. (C) The ratio of half-rising times of SP and *RP*_s_ increases with *G*_MK_ at low uptakes and becomes close to 1 at intermediate and high uptakes. (D) The ratio of the half-falling time of SP and *RP*_s_ decreases with *G*_MK_ at low uptakes and becomes close to 1 at intermediate and high uptakes. The vertical dotted lines indicate the reference value *G*_MK_  =  1.6 nS given in Table 3.

The dose-dependence of the three characteristics of RP and SP in response to two-second square pheromone pulses are shown in Fig. 5. They lead to the following observations. First, the comparison based on the three characteristics of the simulated (*SP*) and measured (*SP*_exp_) values of SP in tip-recording conditions shows that the model reproduces adequately SP_exp_, particularly the fitting to the height curve at high uptakes with 40 compartments (Fig. 4A) is better than with a single compartment (see Fig. 8A in [18]). Second, simulated dose-response curves of RP were determined at four levels along the ORN: tip (*RP*_1_), mid-length (*RP*_20_) and base (*RP*_b_) of the outer dendrite and soma (*RP*_s_). Fig. 5A shows that all these curves have a sigmoid shape as a function of the logarithm of the uptake. All height curves (including that for SP) normalized with respect to their maxima at high uptake are practically superimposed (Fig. 5B). The curves of rising times (Fig. 5C) and falling times (Fig. 5D) are also very similar, except for the rising time at low uptake. So, relative heights, rising times and falling times are strongly dependent on the uptake but can be considered as independent of the location along the ORN. Third, with the present parameter values, the absolute value of *SP* happens to be practically equal to *RP*_s_ at soma. This fortuitous occurrence indicates that the tip-recorded SP reflects very well RP_s_ at all pheromone uptakes. The equality of *RP*_s_ and *SP* is practically independent of the maximum conductances of the depolarizing currents (cationic, Ca^2+^, and Cl^−^ channels; not shown) but strongly depends on the maximum conductance of the repolarizing Ca^2+^- and voltage-dependent K^+^ channel (*G*_MK_; Fig. 6). The amplitude of *RP*_s_ decreases with *G*_MK_ while that of *SP* increases (Fig. 6A) in such a way that their ratio *SP*/*RP*_s_ increases linearly and identically at all uptakes (Fig. 6B). At intermediate and high uptakes, the half-rise times (Fig. 6C) and half-fall times (Fig. 6D) of *RP*_s_ and *SP* are nearly equal for any *G*_MK_ values.

#### 1.3. Sensitivity increase along the cascade

**Figure 7:**
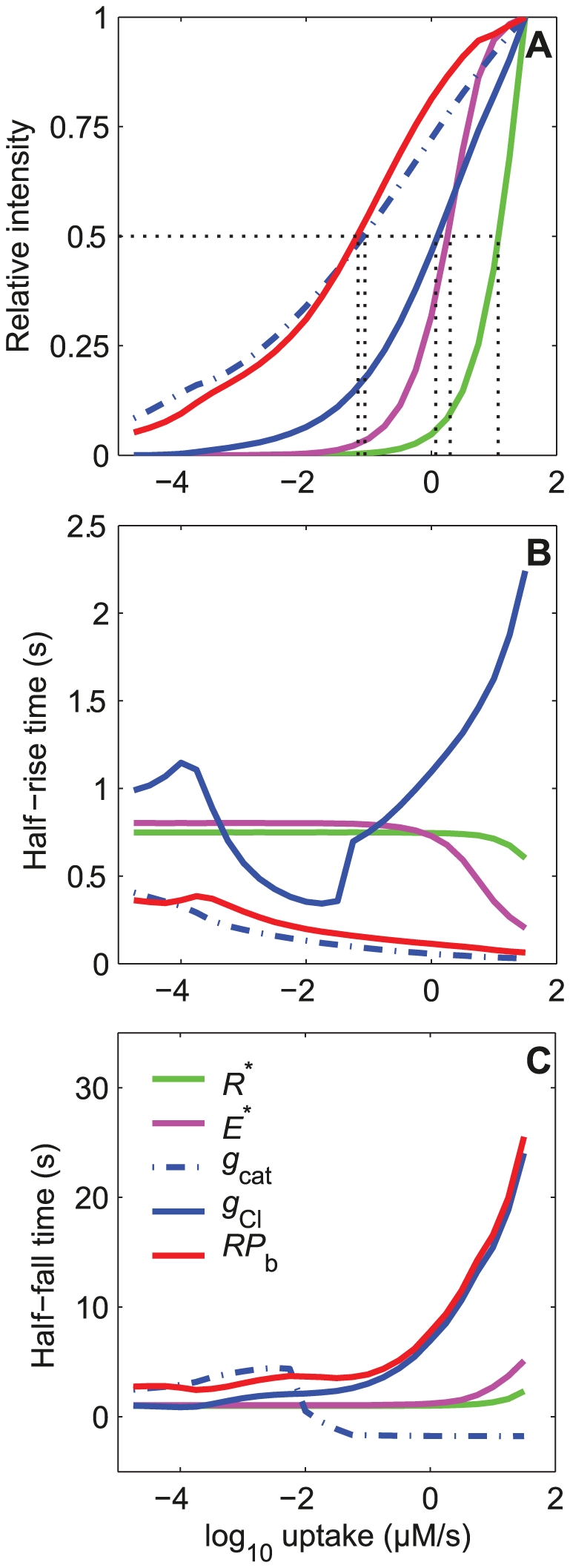
Dose-response characteristics of the major steps in the pheromone transduction cascade. (A) Relative heights of activated pheromone receptor R* (green), effector enzyme E* (magenta), conductance of cationic and chloride channel at the dendrite *g*_cat_ (dash-dotted blue) and *g*_Cl_ (solid blue), and receptor potential at the dendrite base *RP*_b_ (red) as a function of stimulus uptake. The EC_50_'s of R* (11.75), E*(2.0), *g*_cat_ (0.0871), *g*_Cl_ (1.175) and *RP*_b_ (0.069) are indicated (in µM/s). (B) Half-maximum rising times; at EC_50_'s they are 0.70 s (*R* ^*^), 0.68 s (*E* ^*^), 0.08 s (*g*_cat_), 1.15 s (*g*_Cl_) and 0.16 s (*RP*_b_). (C) Half-maximum falling times; at EC_50_'s they are 1.40 s (*R* ^*^), 1.35 s (*E* ^*^), −1.6 s (*g*_cat_), 7.9 s (*g*_Cl_) and 3.70 s (*RP*_b_). It becomes negative for *g*_cat_ when it declines before the end of the 2-s stimulation (falling times are determined from the end of stimulation). The curves for RP_b_ are the same as in Fig. 5. The differential equations and data for R* and E* are the same as in [18], the corresponding parameter values are given in [46] for R* and [49] for E*.

The amplitudes of the input (*R* ^*^) and output (*RP*) variables of the transduction cascade cannot be directly compared because they are not expressed in the same units. So, we compared their normalized amplitudes − ratio of amplitude at any given uptake to the maximum amplitude at high pheromone uptake (10^1.5^ µM/s). The relative dose-amplitude curves of these two variables as a function of pheromone uptake are shown in Fig. 7A. The global amplification of the cascade is apparent as a shift of the *RP* curve to the left of the R* curve. It can be quantitatively expressed by the uptakes that evoke half maximal responses, classically known as efficient concentrations 50 (EC_50_). The EC_50_'s of *R* ^*^ and *RP* are 11.75 and 0.069 µM/s respectively. This corresponds to a global 170-fold (11.75/0.069) increase in sensitivity. It confirms that one of the major functions of the transduction cascade is to amplify weak signals detected by receptors (*R* ^*^) in strong electrical responses (*RP*).

However, in the present model, the gain in sensitivity is not regular along the cascade: the EC_50_'s of the intermediate steps *E* ^*^, *g*_cat_, *g*_Cl_ are 2, 0.0871 and 1.175 µM/s respectively. They indicate that the gain increases in the first two conversions R* to E* (≈6 fold) and E* to *g*_cat_ (23 fold), but decreases in the third conversion *g*_cat_ to *g*_Cl_ (0.07 fold), then increases again in the last conversion *g*_Cl_ to *RP* (17 fold). Overall, the relative height curves of RP and the first (transient) conductance *g*_cat_ are similar (Fig. 7A). This is also true for the rising time (Fig 7B) but not for the falling time of *RP* which is very similar to that of the second (long-lasting) conductance *g*_Cl_ (Fig. 7C). The half-rising time of *g*_Cl_ is not monotonic; it increases except at uptakes between 10^−4^ and 10^−1^ µM/s (Fig. 7B). This is explained by the biphasic kinetics of *g*_Cl_ that presents an initial short bump, corresponding to the peak of the preceding cationic current, followed by a higher and longer wave (this bump and wave are also visible for RP and SP in Fig. 4A–B). The half-rising time is normally determined by the wave, whose rising time is monotonously increasing, except in the range 10^−4^–10^−1^ µM/s where it is determined by the bump, whose rising time is monotonously decreasing (as the corresponding cationic conductance).

### 2. Simplified multicompartmental sensillum model

#### 2.1. Simplification of the pheromone transduction cascade

The transduction cascade converts pheromone concentration in the air (in µM), or better pheromone uptake in the sensillum (in µM/s), to a conductance change of the membrane at the outer dendrite. The total conductance change is the first electrical variable in the cascade and so, a proper level to simplify the whole process and separate the early network of biochemical reactions from the following electrical network. This simplification may benefit to future theoretical analyses and neural network modeling of the olfactory system. To this end, we replaced the set of Ca^2+^, cationic, Cl^−^ and NCX currents at each dendritic compartment *j* with a single current with constant Nernst potential *E*_p_ and variable conductance *g*_p*j*_, where subscript ‘p’ stands for ‘pheromone dependent’. We also removed the K^+^ current of the inner dendrite and soma, which is equivalent to have *G*_MK_  =  0, so that repolarization now results from the leak currents alone.

**Figure 8:**
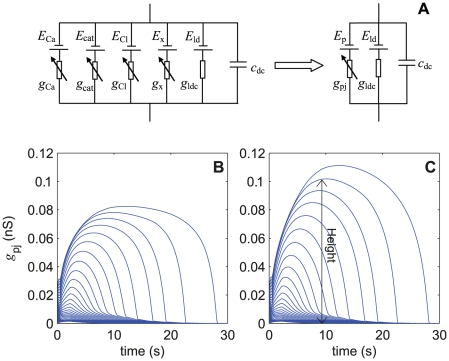
Outer-dendritic compartment and equivalent lumped conductance of the simplified model. (A) Replacement of the original six-branch circuit including four pheromone-dependent conductances (*g*_Ca_, *g*_cat_, *g*_Cl_ and *g*_x_ as described in Fig. 1) with an equivalent three-branch circuit with a single pheromone-dependent conductance *g*_p*j*_ given by eq. (21). (B) Kinetics of *g*_p*j*_ in the first compartment (*g*_p1_) located at the tip of the outer dendrite in response to 2-s square pulses yielding different uptakes regularly spaces by 0.5 log units from 10^−4.75^ to 10^1.5^ µM/s. (C) Idem in the 40^th^ compartment at the base of the outer dendrite (*g*_p40_). Heights of the kinetics were taken at their maximum (indicated with double arrow in next to last uptake).

The replacement of the multiple pheromone-dependent conductances with *g*_p*j*_ was chosen so that the resulting membrane potential be the same in the original model as in the simplified model, at any time, any location along the outer dendrite and any pheromone uptake. The equivalent circuit of a simplified compartment at the ORN outer dendrite is shown in Fig. 8A. The current flowing through *g*_p*j*_ must be the same as the summation of the currents flowing through the four types of channels, i.e. *I*_Ca*j*_ + *I*_cat*j*_ + *I*_Cl*j*_ + *I*_x*j*_. For any compartment *j*, the equivalent conductance *g*_p*j*_ is given by eq. (21) in Methods section.

**Figure 9:**
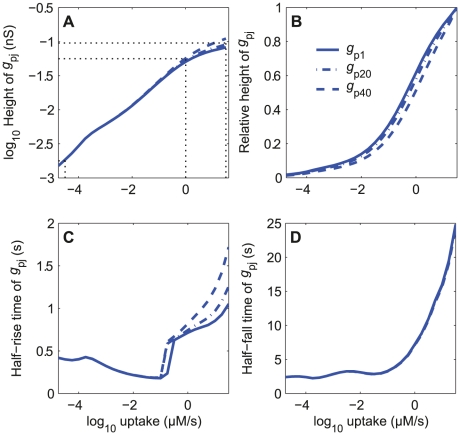
Dose-response characteristics of the simplified model with a single conductance *g*_p*j*_ shown in Fig. 8. Characteristics of *g*_p*j*_ shown for three compartments located at tip (*j*  =  1), mid-length (*j*  =  20) and base (*j*  =  40) of the outer dendrite. (A) Heights (log_10_ *g*_p*j*_) as a function of log_10_ *U*; can be fitted by line log_10_ *g*_p*j*_  =  0.32 log_10_ *U* − 1.28 for *U*≤1. The mean values of *g*_p*j*_ at minimum (*U*  =  10^−4.5^ µM/s), end of the linear growth (*U*  =  1) and maximum (*U*  =  10^−1.5^) uptakes are 1.8×10^−3^, 5.6×10^−2^ and 9.6×10^−2^ nS respectively, as indicated by the dotted lines. (B) Relative heights, *g*_p*j*_/max(*g*_p*j*_) as a function of log *U* at the same locations as in A. (C) Half-rising times. (D) Half-falling times.

For simplicity and keeping close to the two main equilibrium potentials (*E*_cat_ and *E*_Cl_), we took *E*_p_  =  0. Then knowing the total current through the outer-dendritic membrane and the depolarization *V*_id_ − *V*_ed_ from the model and using eq. (21), we determined *g*_p*j*_ at each compartment corresponding to the results given in Fig. 4. Fig. 8B–C shows the kinetics of conductance *g*_p*j*_ obtained for 26 pheromone uptakes at the tip (*g*_p1_, Fig. 8B) and base (*g*_p40_, Fig. 8C) of the dendrite. The characteristics (height, rising and falling times) of conductance *g*_p*j*_, which gives at each uptake *U* the same heights of RP and SP in the single-conductance model as in the multi-conductance model, are shown in Fig. 9. The pairs (log_10_ *U*, log_10_ *g*_p*j*_) are linearly related (Fig. 9A), with only small deviations at very high uptakes. Moreover, the height of *g*_p*j*_ along the outer dendrite, expressed in relative values, remains practically the same at all uptakes (Fig. 9B). This means that the *g*_p*j*_'s yielded by any stimulus, except the largest, are nearly equal across compartments, so that, with a good approximation, the conductance of any compartment can be denoted *g*_p_ (without subscript *j*). Conductance *g*_p_ rises from 1.6×10^−3^ nS at *U*  =  10^−4.75^ µM/s to 0.1 nS at *U*  =  10^1.5^ µM/s. Thus the total pheromone-dependent conductance *G*_p_, sum of all *g*_p*j*_ over the 40 compartments of the outer dendrite, is in the range 6.4×10^−2^ to 4 nS.

The half-rising time (Fig. 9C) at low and intermediate uptakes is determined by the fast cationic current whereas at high uptakes it is given by the slower rising chloride current. The irregularity of the curve (angular point close to 0.1 µM/s) has the same origin as the irregularity of *g*_Cl_ in Fig. 7B; it occurs when the Cl^−^ current overrides the cationic current, that is when the initial conductance peak due to the cationic current becomes less than one-half the maximum (steady state) conductance: then the rising time is no longer that of the cationic current but that of the Cl^−^ current. The half-falling time (Fig. 9D) follows a smooth close to exponential curve.

#### 2.2. Steady state and transient states

The simplified sensillum model involves only electrical components and the pheromone-dependent conductance *G*_p_ is represented by the single ionic channel studied in the previous paragraph. The modeled ORN can be stimulated by a direct change of *G*_p_ bypassing all reactions interposed between the pheromone receptors and the ionic channels. To investigate the electrical properties of the model we used step or square pulses of conductance *G*_p_ taken in the range 6.4×10^−2^ to 4 nS as determined above. The general time-dependent equations for currents (eqs. 20 and 21) and potentials (eqs. 14 with *I*_K_  =  0, 15, 22 and 23) are given in the Methods section.

**Figure 10:**
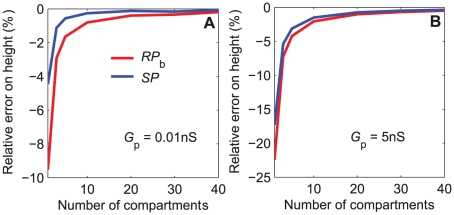
Relative error on steady-state RP and SP depending on number *N* of outer-dendritic compartments. RP at base of the outer dendrite (*RP*_b_) and SP determined in the simplified single-conductance model of Fig. 8. Relative error determined with respect to exact analytical values given by eqs (27) and (34) in Methods section. (A) At low total pheromone-dependent conductance *G*_p_  =  0.01 nS. (B) At high conductance *G*_p_  =  5 nS. The relative error increases with the amplitude of *G*_p_ and decreases with *N*. At high conductance *G*_p_  =  5 nS, it is greater than 22% for *RP*_b_ with one compartment and becomes less than 1% for both *RP*_b_ and *SP* with 40 compartments.

**Figure 11:**
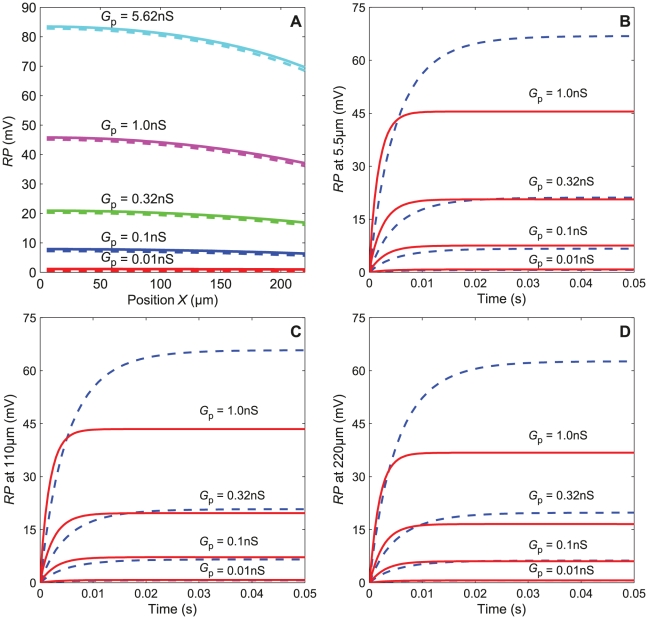
Steady state and kinetics of RP in the single-conductance model. (A) Steady-state RP along the outer dendrite for various values of the total pheromone-dependent conductance *G*_p_. Comparison of simulated values for *N*  =  40 compartments (solid lines) with analytical results given by eqs. (27) to (33) (dashed lines). (B) Kinetics of the transient state *RP*(*t*) close to tip of outer dendrite (*X*  =  5.5 µm, tip is taken as *X*  =  0) in response to step pulses of conductance *G*_p_ of various strengths. Comparison of simulated values with *N*  =  40 compartments (solid lines) with analytical results (dashed lines) given by eqs. (38) to (41) based on an approximation correct only for small RP values. (C) Same at mid-length (*X*  =  110 µm). (D) Same at the base (*X*  =  220 µm).

First, we examined the steady-state responses and compared simulated and analytical results. Vermeulen and Rospars [53] studied analytically a steady-state model of sensillum, identical to the present model, except that it did not include capacitances which play no role at steady state. They determined analytical solutions of the steady-state RP at the base of the outer dendrite (eqs. 24–26 in Methods section) and along its length (eqs. 27–33) and of the steady-state tip-recorded SP (eqs. 34–37). These solutions served here to determine the number *N* of outer dendrite compartments used in the first subsection. The simulated values of *SP* and *RP*_b_ were determined with *N*  =  1 to 40 compartments, in response to step stimulations at a low (0.01 nS) and a high (5 nS) values of the total pheromone-dependent conductance *G*_p_, and compared to the corresponding analytical results (Fig. 10A, B). The steady state response of the model is almost independent of the number *N* of compartments at low and intermediate pheromone uptakes but not at high uptakes. The relative error of the numerical solution with respect to the analytical solution increases with *G*_p_ and decreases with *N*. At very high conductance (*G*_p_  =  5 nS), it is greater than 22% with a single dendritic compartment and less than 1% with 40 compartments. For this reason we chose *N*  =  40. The steady-state RP decreases along the outer dendrite. The difference between *RP*_1_ at the tip (*X*  =  0) and *RP*_40_ at the last compartment (*X*  =  220 µm) increases with *G*_p_ (Fig. 11A). The numerical solution is identical to the analytical solution.

**Figure 12:**
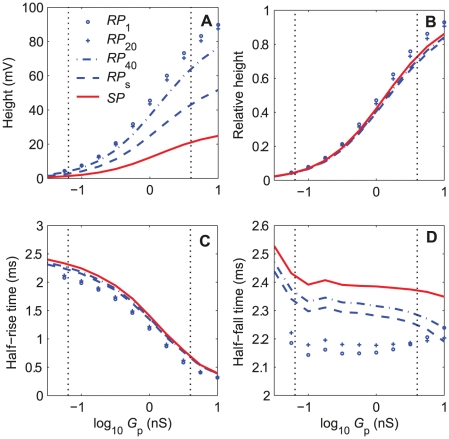
Conductance-response characteristic of RP and SP in the simplified multicompartmental model. Characteristics along the outer dendrite (*RP*_1_, *RP*_20_ and *RP*_40_), at soma (*RP*_s_) and SP in response to 2-s square pulses of conductance *G*_p_. (A) Heights. (B) Relative heights. (C) Half-rising times. (D) Half-falling times. The vertical dotted lines indicate the range of *G*_p_ from 6.4×10^−2^ to 4 nS corresponding to the pheromone uptake rang from 10^−4.75^ to 10^1.5^ µM/s.

Next, we studied the transient-state responses to square pulses of conductance G_p_. The transient time characteristics *τ*_rise_ and *τ*_fall_ of the complete sensillum model depend on the number of compartments. The half-rising time of *RP*_b_ and *SP* increases, while the half-falling time decreases with *N* (not shown). The change is fast when *N* rises from 1 to 10, then the results remain nearly constant. Tuckwell et al. [57] provided an analytical solution for the change in time of the RP following an exponential or instantaneous (step) change of conductance, delivered uniformly at the outer dendrite, in the special case where the depolarization is much smaller than the reversal potential of the permeating ions (here *E*_p_  =  0) (eqs. 38–41). Fig. 11B–D compares the analytical and numerical solutions at the tip, mid-length and base of the outer dendrite in the case of step changes of conductance. Of course, step changes of conductance do not occur with odor stimulation and they are used here only to determine the contribution of the electrical components of the sensillum. They show that the electrical response is fast (rising time ≈5 ms) and that the analytical approximation is better at the tip and remains correct everywhere at better than 16% up to 0.2 nS. The half-falling phase (not shown) is also fast. Fig. 12 illustrates the change of the response characteristics − height, half-rise time and half-fall time − of RP and SP with conductance *G*_p_. It shows that the heights of RP (Fig. 12A) decrease from the tip of the dendrite whereas their relative heights remain practically the same (Fig. 12B). So, at any conductance, SP is about one-third of RP at the tip (*RP*_1_) and one half at the soma (*RP*_s_) which agrees with the result shown in Fig. 6A for *G*_MK_  =  0. Fig. 12C, D confirms that half-rise times (in the range 0.5–2 ms) and half-fall times (2.2–2.4 ms) are much shorter than those of the experimental data (see Fig. 5C and D). This means that the transient states are practically not affected by the pure electrical components of the sensillum. In particular, membrane capacitances have almost negligible effects on the rising and falling phases of the potentials in the actual sensillum.

#### 2.3. Effect of electrical parameters on dose-response characteristics

We analyzed how the various electrical parameters (batteries, conductances and capacitances) of the sensillar circuit affect the characteristics of the conductance-response curves (height, rising and falling times) at the inner dendrite and soma *RP*_s_ and of the tip-recorded *SP*. To this end we compared the characteristics at three intensities of the pheromone-dependent conductance *G*_p_, low (0.1 nS corresponding to *U*≈10^−4.13^ µM/s), medium (1.0 nS, *U*≈0.1 µM/s) and high (10 nS, beyond the range accessible by pheromone stimulation). The results can be summarized as follows:

First, the height of the steady-state potentials is influenced by five parameters (*E*_ls_, *G*_ls_, *E*_a_, *G*_a_, *C*_a_,). The battery at the auxiliary cells *E*_a_ strongly affects the heights of *RP*_s_ and *SP* (Fig. S1A). Removing it decreases the heights of the potentials. The leak battery at the ORN soma *E*_ls_ has similar effects (Fig. S1B). The conductances of the ORN soma (*G*_ls_, Fig. S1C, D) and the auxiliary cells (*G*_a_, Fig. S1E) strongly influence the heights of *SP* and *RP*_s_. Finally the capacitance *C*_a_ of the auxiliary cell membranes has some influence but only at high conductance *G*_p_ (Fig. S1F).

Second, the transient states of *SP* are only weakly influenced by three electrical parameters (*G*_a_, *C*_a_ and *C*_d_) and those of *RP*_s_ by only one (*C*_d_). All other electrical parameters of the sensillum circuit have practically no influence. The conductance of the auxiliary cells *G*_a_ exerts an effect exceeding 10 ms on both rise and fall of *SP* only if it becomes small, less than 1 nS (Fig. S2A, B). The capacitance of auxiliary cells *C*_a_ has an effect exceeding 10 ms on rise and fall of *SP* only for large values, over 50 pF (Fig. S2C, D). Only large changes of the outer-dendrite capacitance *C*_d_ can increase the rising times of *RP*_s_ and *SP* (this effect decreases with *G*_p_; Fig. S2E) and their falling times (effect independent of *G*_p_; Fig. S2F).

Third, to decide whether a given electrical parameter is amplifying the responses or not, we compared the height of the responses of *RP*_s_ at a given *G*_p_ for a small value (often zero) of the parameter (yielding height *RP*_s0_) and for a higher value *x* (yielding height *RP*_s*x*_); if the ratio *f*  =  *RP*_s*x*_/*RP*_s0_ is greater (or smaller) than one, the effect of the electrical parameter is to amplify (or reduce) the ORN response. Note that here we compare heights along the vertical axis instead of comparing sensitivities (EC_50_s) along the horizontal axis as in Fig. 7. Based on this criterion *f* we made the following observations. First, batteries *E*_a_ and *E*_ls_ have a strong amplifying effect that is independent of *G*_p_. The amplification ratio *f_E_*_a_ increases linearly with the absolute value of *E*_a_ (slope is ≈1.6%, Fig. S3A) and the same is true for *f_E_*_ls_ (slope is even steeper, ≈2.8%, Fig. S3B). Second, conductances *G*_a_ and *G*_ls_ have opposite effects that are both dependent on *G*_p_: reducing for *G*_ls_ (especially at low *G*_p_, Fig. S3C) and amplifying for *G*_a_ (especially at low *G*_p_, Fig. S3D). Third, capacitance *C*_d_ (outer dendrite) has no effect; capacitance *C*_s_ has a very weak reducing effect at high *G*_p_ (Fig. S3E), whereas *C*_a_ has a weak amplifying effect (Fig. S3F), especially at high uptakes. We examined also the amplification ratios provided by the conductance-to-voltage conversion from *G*_p_ to *RP*_s_ at different *G*_p_ values. Since *G*_p_ and *RP*_s_ have different units, we compared the relative RP at soma *H*_r_  =  *RP*_s_/max(*RP*_s_) to the relative conductance *G*_r_  =  *G*_p_/max(*G*_p_) which has given rise to it. With the standard parameter values, the corresponding ratio *f*_r_  =  *H*_r_/*G*_r_, is equal to ≈8.4 at very weak stimulation (*G*_p_  =  0.01 nS) then declines to 1 at strong stimulation (*G*_p_  =  10 nS). Simulation results show that most parameters have very weak (*G*_a_, *C*_d_, *C*_s_) or no effect at all (*E*_a_ and *E*_ls_) on *f*_r_. The only exceptions are *G*_ls_, which has a strong reducing effect from ≈8.4 for *G*_ls_  =  1.5 nS to 5.2 for 5 nS (Fig. S4A), and *C*_a_, which displays a relatively weak reducing effect from ≈8.4 for *C*_a_  =  3.5 pF to 6.8 for *C*_a_  =  300 pF (Fig. S4B).

#### 2.4. Effect of geometrical parameters on dose-response characteristics

**Figure 13:**
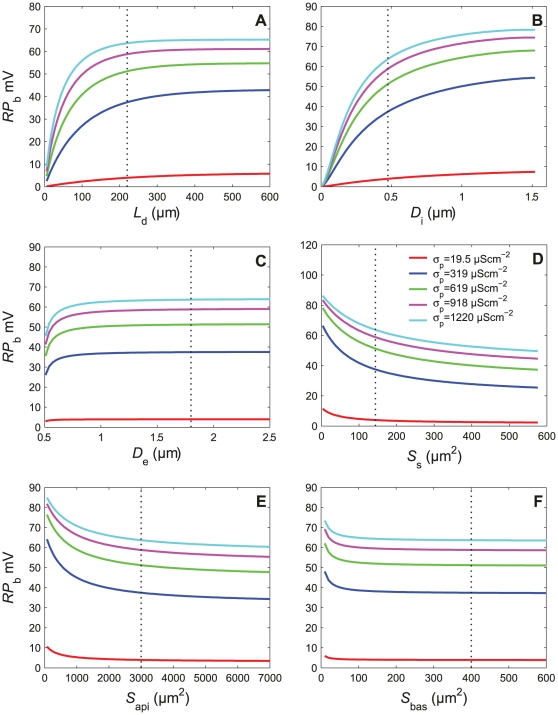
Effects of geometric parameters on the steady-state RP at the base of the outer dendrite. *RP*_b_ shown in response to different values of the pheromone-dependent conductivity *σ*_p_ (in µScm^−2^). (A) Length of outer dendrite *L*_d_. (B) Diameter of outer dendrite *D*_i_. (C) Diameter of hair lumen *D*_e_. (D) Area of inner dendrite and soma *S*_s_. (E) Area of apical membrane of auxiliary cells *S*_api_. (F) Area of basal membrane of auxiliary cells *S*_bas_. The vertical dotted lines indicate the biologically realistic parameter values given in Tables 1 and 2.

The resistance and capacitance of various sensillum parts depend directly on the area of the corresponding membranes. Therefore, the effects of electrical parameters can be analyzed in a less abstract way via the geometric characteristics of the sensillum. Since the electrical parameters exert a minor influence on the transient states, we examined the effects of the geometric parameters on the steady state only. Using eqs. (24) – (26), we calculated the steady-state value of the receptor potential at the base of the outer dendrite *RP*_b_ as a function of various geometric parameters in response to five different values of pheromone-dependent conductivity *σ*_p_ (in S.cm^−2^). Fig. 13 shows that *RP*_b_ increases monotonously with the length *L*_d_ and diameter *D*_i_ (hence area) of the outer dendrite and diameter and *D*_e_ of the hair lumen, and decreases monotonously with the areas *S*_s_ of the inner dendrite and soma, and *S*_api_ and *S*_bas_ of the apical and basolateral membranes of the auxiliary cells. For most parameters, asymptotic values of *RP*_b_ are reached for the actual value of the parameter found in *Antheraea* (shown as dotted vertical lines in Fig. 13).

## Discussion

In the following discussion we distinguish the “molecular parameters” that describe membrane proteins (e.g. ionic channels) and diffusible modulators (e.g. Ca^2+^) and “cellular parameters” that describe the geometrical and electrical properties of the sensillum.

### 1. Molecular bases and assumptions

The sensillum model studied here is based on a detailed model of the biochemical processes generating the transmembrane receptor potential and the transepithelial sensillar potential (Fig. 3). The main conclusions drawn from simulating the model are: (i) As shown previously [18], it accounts for the wide dynamic range of the pheromonal ORN, for the short rising time of the RP and for its slow decline after the end of the stimulation. This is noteworthy because extracellular reactions alone cannot account for the falling time of SP at uptake larger than 1 µM/s [41], thus contradicting Kaissling's suggestion [41], [45] that intracellular events play no role in determining RP kinetics. (ii) The cationic and the Cl^−^ currents are the two main depolarizing currents. (iii) They play different roles depending on the pheromone concentration: the cationic current is the main depolarizing current at low concentration, the Cl^−^ current the main one at intermediate and high concentrations. (iv) The Ca^2+^ extrusion mechanism depends on potential which supports the hypothesis of a Na^+^-Ca^2+^ exchange (NCX). However, three assumptions in the model – the involvement of G-proteins, the depolarizing role of the Cl^−^ current and the repolarizing role of the K^+^ current – are still uncertain and deserve specific discussions.

First, in recent years the requirement for G-proteins in ORNs has been much debated (reviewed in [18], [19], [20], [21]). Two studies in *Drosophila* found that a ubiquitously expressed coreceptor, OR83b, alone or coupled to ORs, form a ligand-gated ion channel, but one of these studies conclude that G-proteins are involved in olfactory responses [34] whereas the other report claims that they play a negligible role [33]. Although some studies support the involvement of G-proteins [58], [59], recent results, also in *Drosophila*, using single-sensillum recordings, do not confirm this involvement in ORNs [35]. Whether the same conclusion holds true for moth pheromonal ORNs is not known, although some evidence exists for the involvement of Gq proteins and PLC in this cascade [14], [60]. However, as far as the quantitative aspects studied in this paper are concerned, whether the pathway is metabotropic (via G-proteins) or ionotropic (without G-proteins) is not essential for two reasons. First, in both cases ORs trigger Ca^2+^-permeable cationic channels, either indirectly (via G-proteins and PLC effectors) or directly. Second, in the present model, although based on the metabotropic pathway, the amplification from OR to PLC effectors accounts for only 3.5% of the total amplification (Fig. 7; [49]). This means that removing the metabotropic processes (G-proteins, PLC and second messengers) from the model, i.e. replacing the original metabotropic model by a ionotropic model would not alter much the quantitative results obtained, provided the cationic current keeps the same transient kinetics in both models.

Second, it is not known whether the Ca^2+^- and voltage-dependent K^+^ current is located in the outer or inner dendrite. If it is in the outer dendrite, it cannot be repolarizing because the equilibrium potential of K^+^ ions there is close to 0 mV. If it is in the inner dendrite, its modulation by Ca^2+^ cannot be direct because of the slow diffusion of this ion. The simplified model is informative here because it shows that leak currents are sufficient to repolarize the ORN, so that the K^+^ current is not needed for this function.

Third, it remains uncertain whether the Ca^2+^-dependent Cl^−^ current is depolarizing, as in vertebrates, or repolarizing because the equilibrium potential of Cl^−^ (*E*_Cl_) is unknown. In a recent experimental study, we showed that reducing the extracellular Ca^2+^ [39] or Cl^−^ concentration [36] does not significantly modify the SP amplitude but slows down its return to the resting level. Although this effect is consistent with a repolarizing function, it does not fully exclude the possibility that the decrease in extracellular Cl^−^ concentration increases the depolarizing current and so results in the observed lengthening of the response. The uncertainty can be settled by measuring the Cl^−^ concentration in the sensillar lymph or blocking the Cl^−^ channel. The consequences of a repolarizing Cl^−^ current would be a more efficient repolarization than with leak currents alone. However, the long-lasting Cl^−^ of the model would have to be replaced with another depolarizing current with the same long-lasting kinetics. Then, in order to account for the measured properties of the SP, the sum of all depolarizing currents, especially of the transient cationic current and of this new depolarizing current, should remain the same as the lumped receptor current predicted by the simplified model with a single pheromone-dependent conductance. If the mechanism is ionotropic and a single depolarizing cationic current exists, it would have to behave differently from ionotropic synaptic receptors that usually close immediately after the neurotransmitter removal; this suggests that the insect ORs might stay active after the ligand has been removed [61].

In summary, although quantitative modeling based principally on SP measurements of the kind considered here cannot distinguish between the ionotropic and metabotropic pathways and between the depolarizing and repolarizing functions of the Cl^−^ current, it provides precise information for example on the relative strength of the RP and the SP, or on the kinetics and intensities of the currents. These results, at least in principle, give the possibility to differentiate between the different hypotheses. A good example is provided by the DAG-gated cationic current. It follows from the previous discussion that its kinetics is a key aspect to resolve the intricacies of the cascade. We have considered this current as transient because the production of IP_3_ and therefore DAG, in moth pheromonal ORNs has been shown to be transient in stop flow experiments [24]. If the main transduction pathway of this ORN were ionotropic as in the fruit fly, the production of IP_3_ (and DAG) would not be part of the pathway and there would be no compelling reason to consider the cationic current as transient. Then, provided its intensity turns out to be large enough, this long-lasting cationic current could be identified with the lumped current of the simplified model and so account quantitatively for the SP properties.

### 2. Molecular parameters

Although the present model is based on several qualitative assumptions at the molecular level, not all aspects of the model are dependent on these assumptions. A recurrent problem concerns the validity of estimating parameter values from experimental data using a single point model, or more generally of comparing values from models of different degrees of geometrical realism. It is standard in neuron modeling to use models with only a few compartments or even reduced to a single point (e.g. [62], [63]). The olfactory sensillum is a good example for studying this problem because it is a small multi-cell organ with spatially extended neurons. Both aspects (several cells, long dendrite) significantly influence the experimentally measured variable, the transepithelial sensillar potential (SP).

We have compared two models of this system, both with a single ORN within the sensillum. The first model involved only 3 compartments, two for the ORN (outer dendrite and inner dendrite plus soma) and one for the auxiliary cells [18]. The second model, analyzed in the present paper, takes into account the spatial extension of the outer dendrite, which is divided in *N*  =  40 compartments, and keeps unmodified the two other compartments (inner dendrite plus soma and auxiliary cells). All molecular details were kept identical in both models, except that concentrations of proteins and diffusible modulators, currents and potentials were computed independently in each outer-dendritic compartment of the second model.

Table 3 shows that only 8 of the 38 molecular parameters were modified to adapt the model with a single outer-dendritic compartment to the model with forty outer-dendritic compartments. Of these 8 parameters, five (*s*_M_, *G*_MCa_, *G*_Mcat_, *G*_MCl_, *G*_MK_) control the maximum amplitude of the ionic currents. In all cases this amplitude had to be reduced. Because all these parameters are given for the whole outer dendrite, this reduction comes in addition to the division by *N* of the conductances for the whole outer-dendrite to get conductances in each compartment. The three other modified parameters were the Hill coefficients of the two main currents (*n*_cat_, *n*_Cl_) and the equilibrium potential of the NCX pump (*E*_x_); they can be considered as secondary adjustments. The only current whose maximum amplitude was not modified was Ca^2+^ extrusion.

The practical consequence of this finding is encouraging. It means that the long and computer-demanding process of fitting parameter values to the experimental data can be done in two steps. In a first step, a preliminary solution can be obtained with the simplified single compartment model. In a second step, this solution can be refined by modifying essentially the maximum conductances of the multi-compartmental model while keeping all other parameter values unchanged.

The rising and falling times of RP and SP are practically the same at all pheromone stimulations. A divergence appears only at weak stimulations (below 10^−3^ µM/s) where the rising time of RP at soma is shorter than that of SP (Fig. 4C).

### 3. Cellular parameters

In previous work we studied analytically the steady-state properties of this system [52], [53], [64] or the transient state with restrictive assumptions [57]. Here we examined numerically both the steady and transient states without such assumptions and with realistic values of the parameters. For studying the global electrical properties, it is practical to replace the full molecular model with a simplified equivalent model. At the outer dendrite, the whole set of pheromone-dependent conductances (*g*_Ca_, *g*_cat_, *g*_Cl_, *g*_x_) shown in Fig. 3 was replaced with a single conductance *g*_p*j*_ in each compartment, while keeping the leak conductance *g*_ld_ (Fig. 8). The lumped pheromone-dependent conductance requires the choice of the equilibrium potential of the permeating ions; we took *E*_p_  =  0, in agreement with patch clamp observations of the reversal potential of the receptor current [26] and the fact that this current is mostly carried by cations (*E*_cat_  =  0) and chloride ions (*E*_Cl_  =  −11.5 mV in our model). At the inner dendrite and soma, the pheromone-dependent conductance *G*_K_ was removed while keeping the leak conductance *G*_ls_.

The resulting simplified model accounts as well as the detailed model for the experimental SP measurements. A negative consequence of this fact is, as noted above, that SP measurements alone cannot help to decide the molecular and ionic mechanisms active in the system. In particular, the simplified model shows that the leak conductances (*g*_ld_ and *g*_ls_) could be sufficient to repolarize the neuron after a pheromone stimulation. So, the role of Cl^−^ and K^+^ currents must be resolved by other experiments. A positive consequence, illustrated below, is that many significant properties of the pheromone-sensitive ORN and sensillum can be studied independently of these underlying mechanisms.

The outer dendrite is relatively compact: its total (cut) length (220 µm, Table 1) is about ½ space constant (λ  =  450 µm). For pheromone uptakes *U* between 10^−4.5^ and 1 µM/s, the logarithm of the height of the pheromone-dependent conductance *g*_p*j*_ is the same whatever the location of compartment *j* along the dendrite and depends linearly on the logarithm of *U* (Fig. 9A). The maximum overall conductance *G*_p_≈*Ng*_p_ at 10^1.5^ µM/s (4 nS) is about nine-fold the resting membrane conductance (0.44 nS). On the same range of uptakes, the RP at soma varies between 0 and 28 mV. When directly stimulated with square pulses of pheromone-dependent conductance *G*_p_, the maximal half-rise and half-fall times are practically the same for RP and SP (2.5 ms, Fig.12C, D) and less than one-third the membrane time constant (7.5 ms). These transient times are much longer with pheromone stimulation, the half-rise times of RP and SP being in the range 0.05 to 0.4 s (Fig. 5C) and the half-fall times in the range 2.5 to 25 s (Fig. 5D), that is 20 to 160 and 860 to 8600 times longer respectively. Clearly the transient times of RP and SP can only be explained by molecular mechanisms, not by the electrical conductance-to-RP conversion mechanisms. Previous simulations reported in Gu et al. [18] showed that most of the rising (66–92% depending on uptakes) and falling times (82-90%) result from the extracellular translocation and deactivation of pheromone molecules. We determined these percentages by stimulating directly the modeled cascade with square pulses of activated receptor R* instead of square pulses of pheromone, so removing the time taken by the perireception and reception processes.

With the assumption *E*_p_  =  0, we showed analytically [53] and confirmed numerically here, that the ratio of the tip-recorded SP to the RP at the base of the outer dendrite is independent of the values of the batteries and depends only on the ratio of the resistances per unit length of the sensillum lymph and the intradendritic medium *r*_e_/(*r*_e_ + *r*_i_), the ratio *a* of the auxiliary-cell resistance to the total resistance of the nonsensory part *R*_a_/(*R*_ls_ + *R*_a_), and the length *L*_d_ of the dendrite. With the present parameter values the ratios of the amplitudes *SP*/*RP*_b_ and *SP*/*RP*_s_ are about one third and one half respectively (Fig. 12A; in the complete model these ratios are 0.5 and 1.0 respectively, see Fig. 5A). This ratio remains the same whatever the stimulation (Fig. 12B), so at steady state SP is proportional to RP. This conclusion remains true in the complete model (Fig. 6), i.e. whatever the depolarizing currents (with a single pheromone-dependent conductance or not) and repolarizing currents (with only leak currents or not). It is in good agreement with the experimental observation that SP is proportional to RP [65].

### 4. Insect sensilla in an engineering perspective

In insects, olfactory sensilla come in very different shapes and sizes, both in different species and in the same species. For example, the flat sensilla placodea of Hymenoptera contrast with the hair-like sensilla of Lepidopera. Also, in Lepidoptera, the long sensilla trichodea housing pheromone-sensitive ORNs contrast with the short sensilla basiconica housing ORNs sensitive to allelochemicals (plant and other non-pheromonal odors). These variations affect primarily the geometrical characteristics of the sensillum listed in Table 1 like the length *L*_d_, and diameters *D*_i_, *D*_e_ characterizing the outer dendrite and hair lumen. If the shape of the various sensillum components is not simple, for example if the outer dendrite and surrounding cuticle is not cylindrical, membrane areas (*S*_d_, *S*_s_,) and compartment volumes (*V*_d_, *V*_e_) may be more appropriate descriptors. For simplifying this discussion we will consider only variations in size.

Most physiologically-relevant properties of sensilla, i.e. those influencing their coding properties, ultimately depend on their internal geometry. The reason is that the geometry is the main way to modify the electrical properties. For example, increasing the diameters of the outer dendrite and hair lumen (hence the volumes) decreases the resistances per unit length (*r*_d_, *r*_e_, *r*_i_). Similarly, increasing membrane areas (*S*_d_, *S*_s_, *S*_api_ and *S*_bas_) increases their capacitances (*C*_d_, *C*_s_, *C*_a_) but decreases their resistances (*R*_ld_, *R*_ls_, *R*_a_) as shown by the relationships given in the right column of Table 2. Although the final physiological effects depend in a complex way on these changes, they can be summarized briefly: increasing the area of the outer dendrite or the diameter of the hair lumen increases RP whereas increasing the areas of the other parts (inner dendrite and soma, apical and basolateral membranes of auxiliary cells) has the reverse effect (Fig. 13). It is also noteworthy that for the actual values of *L*_d_, *D*_e_, *S*_api_ and *S*_bas_ observed in *Antheraea*, the horizontal asymptotic part of the RP curves is reached (or almost reached), so that they could be decreased without significantly decreasing the RP. This is not the case for *D*_i_ and *S*_s_: if the outer-dendrite diameter was greater or the inner dendrite and soma were smaller, RP would be greater. Although these effects on RP amplitude may contribute to explain the long hairs and relatively compact internal parts of the moth pheromone sensillum, the fact that the geometric parameters *D*_i_ and *S*_s_ are not optimum (i.e. close to the value yielding the asymptotic RP) means that other constraints mould the shape and size of the sensillum. Although the reason why the area *S*_s_ of the inner dendrite and soma is suboptimal is unclear, one can speculate that the diameter *D*_i_ of the outer dendrite is limited by the metabolic cost of the transduction machinery that would result from a larger membrane area.

## Methods

### 1. Equations of the multicompartmental and multichannel sensillum model

As schematized in Fig. 2, each outer-dendritic compartment includes a circuit representing a cylinder segment of membrane, with six parallel branches: the capacitive current (membrane capacitance *C*_d_), the leak current (conductance *g*_ldc_ and battery *E*_ld_, the subscript ‘d’ stands for ‘dendrite’ and ‘c’ for ‘compartmental’) and the four pheromone-dependent currents (conductances *g*_y_ and batteries *E*_y_ described below). The inner dendrite and soma compartment includes a three-branch circuit with capacitive current (membrane capacitance *C*_s_), leak current (conductance *G*_ls_ and battery *E*_ls_, the subscript ‘s’ stands for ‘soma’) and Ca^2+^- and voltage-dependent potassium current (conductance *G*_K_ and battery *E*_K_). The three auxiliary cells are lumped in a circuit composed of two branches with a capacitive current (membrane capacitance *C*_a_) and a leak current (conductance *G*_a_ and battery *E*_a_, the subscript ‘a’ stands for ‘auxiliary’). The sensillar lymph which bathes the outer dendrite is described by a series conductance *g*_ec_ (subscript ‘e’ stands for ‘extracellular’). The intracellular medium (subscript ‘i’) is also described by a series conductance *g*_ic_. Pheromone-dependent conductances *g_y_*(*t*) and *G*_K_(*t*), current intensities *I*(*t*) and membrane potentials *V*(*t*) depend on time. For simplifying the notations, variable *t* is omitted wherever possible.

#### 1.1. Equations for currents

In each compartment of the outer dendrite, five types of ionic channels are taken into account. The IP_3_-gated Ca^2+^ current *I*_Ca_, DAG-gated cationic current *I*_cat_, intracellular Ca^2+^-gated chloride current *I*_Cl_ and Na^+^/Ca^2+^ exchange (NCX) current *I*_x_ at the *j*th compartment of the outer dendrite are described by
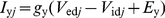
(1)where subscript ‘y’ represents Ca^2+^, cationic, chloride and NCX currents respectively, *V*_ed*j*_ is the extracellular potential (in sensillum lymph) and *V*_id*j*_ is the intracellular potential (within the outer dendrite). In a uniformly stimulated membrane, conductance *g*_y_ is a constant, independent of compartment *j*, described by
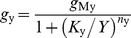
(2)where *g*_My_ is the maximum ionic conductance of the channels for each compartment at the outer dendrite, *Y* the concentration of the agonist Y of the channels (the agonists for *I*_Ca_, *I*_cat_, *I*_Cl_ and *I*_x_ are IP_3_, DAG, Ca^2+^ and Ca^2+^ respectively), *K*_y_ the concentration of Y producing their half-maximal conductance (EC_50_). Eq. (2) is basically the same as eq. (2) in Gu *et al*. [18] except for *g*_My_  =  *G*_M*j*_/*N* since the dendrite in the present model is divided into *N* compartments. Here, we suppose that these channels are uniformly distributed at the outer dendrite. For channels modulated by an antagonist Z (Ca^2+^-calmodulin for Ca^2+^ and cationic channels, and protein kinase C for Cl^−^ channels), *K*_y_ is given by 
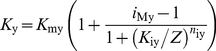
(3)where *K*_my_ is the EC_50_ in absence of Z (then *K*_y_  =  *K*_my_ is at its lowest value), *K*_iy_ is the concentration of Z producing half-maximal inhibition (IC_50_) and *i*_My_ is maximum inhibition at high concentration of Z (then *K*_y_  =  *K*_my_ *i*_My_ is at its highest value).

The leak current at the *j* ^th^ compartment is described by
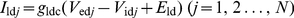
(4)where *g*_ldc_ is the constant leak conductance at each dendritic compartment (inverse of the membrane specific resistance at rest). Finally, the extracellular longitudinal currents are
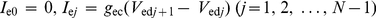
(5)


At the inner dendrite and soma compartment two types of ionic channel are considered, the K^+^ current *I*_K_ (agonist Ca^2+^, no antagonist, voltage-dependent) and the leak current *I*_ls_. Their intensity is given by
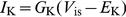
(6)

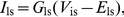
(7)where conductance *G*_K_ depends on the Ca^2+^ concentration (*Ca*) and the membrane potential (*V*) and conductance *G*_ls_ is constant. For *G*_K_, we used a modified version of eq. (2)
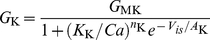
(8)where *G*_MK_, *K*_K_ and *A*_K_ are constants.

The intracellular longitudinal current (*I*_i*N*_) flowing from the *N* ^th^ outer dendrite compartment to the inner dendrite and soma compartment and the extracellular longitudinal current (*I*_e*N*_) flowing from the auxiliary cells to the *N* ^th^ outer dendrite compartment is
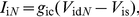
(9)

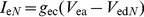
(10)


The leak current at the auxiliary cells is 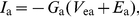
(11)


#### 1.2. Differential equations for potentials

Differential equations for the potentials are derived from Kirchhoff’s current law. The outer dendrite is divided in *N* isopotential compartments. In each compartment *j*, with *j*  =  1, 2, … *N*, the internal (*V*_id*j*_) and external (*V*_ed*j*_) potentials obey the following differential equations
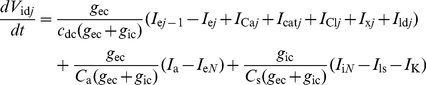
(12)

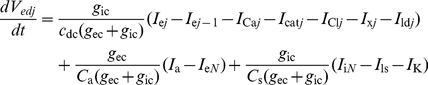
(13)


At the inner dendrite and soma:
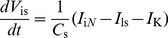
(14)


At the auxiliary cells
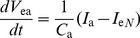
(15)


This system of differential equations was integrated numerically with the Matlab ode45 solver (The Mathworks, Natick, USA).

#### 1.3. Receptor potential and sensillar potential

With pheromone stimulation starting at time 0, RP at time *t* at any point along the neuron is equal to the difference of intracellular potentials during stimulation (time *t*) and at rest (time 0). So, RP at the *j*th compartment of the dendrite at time *t* is
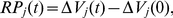
(16)


where 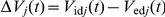 is the transmembrane potential of segment *j* at time *t*. Similarly, RP at the base of the outer dendrite at time *t* is
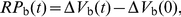
(17)


where 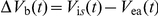 is the transmembrane potential at the base of the outer dendrite at time *t*. Finally, RP at the ORN inner dendrite and soma is 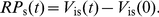
(18)


The tip-recorded SP is the difference between the external potential of the first compartment during stimulation at time *t* and at rest at time 0: 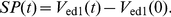
(19)


### 2. Equations of the simplified sensillar model with single pheromone-dependent channel

The total transmembrane pheromone-dependent current flowing through the Ca^2+^, cationic, Cl^−^ and NCX channels in the *j*th compartment of the outer dendrite can be replaced by a single equivalent current *I*_p*j*_  =  *I*_Ca*j*_ + *I*_cat*j*_ + *I*_Cl*j*_ + *I*_x*j*_, where the subscript ‘p’ stands for pheromone-dependent. The corresponding circuit branch involves a lumped battery *E*_p_ (the same in all compartments) and a lumped conductance *g*_p*j*_ replacing the ionic conductances *g*_Ca_, *g*_cat_, *g*_Cl_ and *g*_x_. All equations are the same as above except those that involve the pheromone-dependent conductances. From eq. (1) the equivalent current can be written 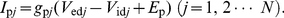
(20)


Therefore, the equivalent conductance is
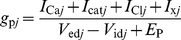
(21)


Note that *g*_p*j*_ depends on compartment *j*, unlike *g*_Ca_, *g*_cat_, *g*_Cl_ and *g*_x_.

The differential equations (12) for the intracellular potential and (13) for the external potential (in the sensillum lymph) become, with *N* the number of compartments and *j*  =  1, 2, … *N*, 
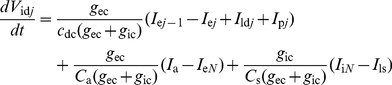
(22)

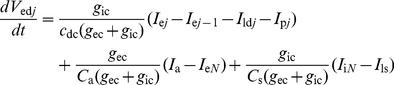
(23)


### 3. Equations for the steady state based on the cable equation

In this section the outer dendrite is described by a cable [12], [52], [53]. This model is equivalent to the multicompartmental model considered in the previous section with an infinite number of cylindrical compartments. Axial symmetry is assumed and we only consider one space variable, the distance *X* along the cylinder, taking the tip of the outer dendrite as origin (*X*  =  0). To have simpler equations, distance *X* and length *L*_d_ of outer dendrite are expressed in membrane space constants, *x*  =  *X*/*λ* and *l*_d_  =  *L*_d_/*λ.* Notations are the same as those used in the compartmental description, except for the “per compartment” parameters (*c*_dc_, *g*_ec_, *g*_ic_, *g*_ldc_) that are now replaced with corresponding “per unit length” parameters, denoted without the subscript ‘c’: *c*_d_, *g*_e_, *g*_i_, *g*_ld_ (and the corresponding resistances *r*_e_, *r*_i_, *r*_ld_).

#### 3.1. Steady-state receptor potential at the base of the outer dendrite

From eq. (17) the steady-state receptor potential *RP*_b_ at *x*  =  *l*_d_ is 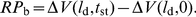
(24)where Δ*V*(*l*_d_,*t*_st_)  =  *V*_i_(*l*_d_,*t*_st_) − *V*_e_(*l*_d_,*t*_st_) and Δ*V*(*l*_d_,0) are the transmembrane potentials at the base of the outer dendrite at steady state (at any time *t*_st_ when steady-state is established) and at rest (at time 0) respectively. These potentials are given by eqs. (6) and (8) in Vermeulen and Rospars [53]. With the present notations they can be written
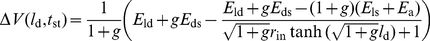
(25)

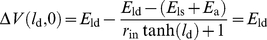
(26)where *r*_in_ (dimensionless ratio of resistances) is given in Table 2 and *g* (dimensionless pheromone-dependent conductance) is given in Table 4.

**Table 4:** Main time-dependent variables from stimulus to responses.^a^

	Variables	Symbol	Unit	Reference or explanation
Pheromone	Concentration in air	*L*_air_	µM	*L*_air_ = *U*/*k*_i_ with *k*_i_≈10^4^ s^-1^ [47]
	Uptake in sensillum	*U*	µM/s	[45]
Complete model	Concentration of activated pheromone receptor	R*	µM	[49]
	Concentration of activated effector enzyme (PLC)	E*	µM	[49]
	Conductance of channel y b	*g*_y_	nS	Eq. (2)
	Current borne by channel y b in compartment *j*	*I*_y*j*_	pA	Eq. (1), [18]
	Transmembrane potential	*ΔV_j_*	mV	Eqs. (16)–(17)
	Receptor potential	*RP_j_*	mV	Eq. (16), Figs. 4C, 5
Simplified model	Total receptor current in compartment *j*	*I*_p*j*_	pA	Eq. (20)
	Pheromone-dependent conductance in compartment *j*	*g*_p*j*_	nS	Eq. (21), Figs. 8 and 9
	Pheromone-dep. conductance of whole outer dendrite	*G*_p_	nS	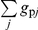 ≈*Ng*_p1_
	Pheromone-dependent conductivity	*σ*_p_	Scm^−2^	*σ*_p_ = *G*_p_/π *L*_d_ *D*_i_
	Pheromone-dependent conductance per unit length	*g*_p_	Scm^−1^	*g*_p_ * = σ*_p_ π*D*_i_
	Dimensionless pheromone-dependent conductance	*g*	*g*_ld_	*g = r*_ld_ *g*_p_ or *g* = *ρ*_ld_ *σ*_p_
	Transmembrane potential	*ΔV*(*x*)	mV	Eqs. (24) – (27)
	Receptor potential	*RP*(*x*)	mV	Fig. 11
Both models	Sensillar potential	*SP*	mV	Figs. 4B, 5, 10, 12

a All variables listed depend on pheromone concentration *L*_air_ and therefore on time *t*. *L*_air_ is assumed to be applied uniformly over the sensillum hair. Variables *U*, R*, *g*_y_ are independent of the location (compartment *j* or abscissa *x*) along the outer dendrite but not variables *I*, *ΔV* and *RP*.

b Subscript ‘y’ refers to Ca^2+^, cationic, Cl^−^ or K^+^ channels or to Na^+^-Ca^2+^ exchanger.

#### 3.2. Steady-state receptor potential along the outer dendrite

From eqs. (16) – (18) the receptor potential at distance *x* (expressed in membrane space constant) from the tip of the outer dendrite *RP*(*x*,*t*_st_), with 0≤*x*≤*l*_d_, is
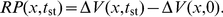
(27)where Δ*V*(*x*,*t*_st_) and Δ*V*(*x*,*0*) are the transmembrane potentials at steady state and at rest respectively. The intracellular membrane potentials and the extracellular potentials are given by eqs. (11) and (12) respectively in Vermeulen and Rospars [53]. With the present notations they can be written as 
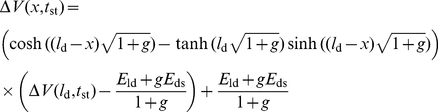
(28)

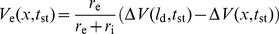
(29)


These potentials at resting state can be obtained by taking *g*  =  0:
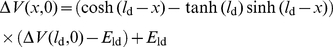
(30)

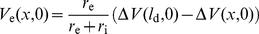
(31)

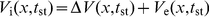
(32)

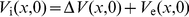
(33)where Δ*V*(*l*_d_,*t*_st_) and Δ*V* (*l*_d_,0) are given by Eqs. (25) and (26).

#### 3.3. Sensillar potential at steady state

From eq. (19) the tip-recorded sensillar potential at steady state is
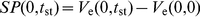
(34)where *V*_e_(0,*t*_st_) and *V*_e_(0,0) are the potentials recorded at the tip of the hair at steady state (corresponding to time *t*_st_) and at rest (corresponding to time 0) respectively. These membrane potentials are given by eqs. (16) and (17) in [53]. With the present notations they can be written as
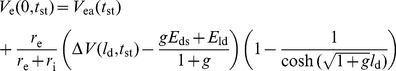
(35)

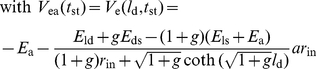
(36)

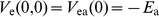
(37)where *g*, *l*_d_ are defined above. Parameters *r*_e_, *r*_i_, *a*, *r*_in_ are given in Table 2. *E*_A_, *E*_rN_, *E*_rS_ and *E*_S_ in eqs. (5) – (8), (11), (12), (16) and (17) of [53] correspond to −*E*_a_, −*E*_ls_, −*E*_ld_ and *E*_p_ respectively, in the present paper.

### 4. Equation for the transient state

Tuckwell et al. [57] analyzed the depolarization *V*(*X*, *t*) at any point *X* and time *t* along a cable of length *L* with both ends sealed representing the ORN. The outer dendrite containing the receptor-potential generating mechanisms extends from *X*  =  0 to *X*  =  *L*_d_, with *L*_d_ < *L*. Again, to simplify equations, distance *X* and lengths (*L*_d_, *L*) are measured in membrane space constants (*x*  =  *X*/*λ*, *l*_d_  =  *L*_d_/*λ*, *L*'  =  *L*/*λ*) and time *t* in time constants (*t*'  =  *t*/*τ*). For a uniform change of conductance 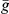 (measured in membrane conductance at rest) along the outer dendrite, starting at time *t*'  =  0 and obeying function 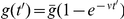, the depolarization *V*(*x*,*t*') could be obtained analytically only in the special case *V* << *E*_p_, where *E*_p_ is the reversal potential of the permeating ions, as
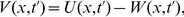
(38)where 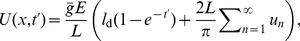
(39)with 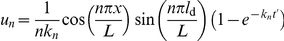 and 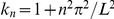,
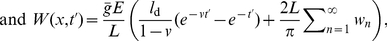
(40)


with 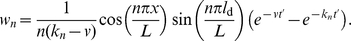


In the case of a step function 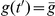, the solution simplifies to



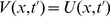
(41)


### 5. Parameter estimation

The unknown parameters of the multicompartmental and multichannel sensillum model were estimated as explained previously [18]. First, we imposed that each parameter be in a physiologically acceptable range of values compatible with the properties of our qualitative model of transduction. Second, we considered a parameter set *θ* as acceptable if the predicted kinetics of SP were close to the experimentally measured kinetics at all uptakes. For checking this condition, we minimized a cost function based on the three response characteristics, height (*H_i_*), rising time (*τ*_rise,*i*_) and falling time (*τ*_fall,*i*_) at a series of uptakes *i*. The differences, *ΔH_i_  =  |H_i_* - *Ĥ_i_|*, between the values *Ĥ_i_* predicted by the model for a given set *θ* and the experimental values *H_i_*, were determined. The differences *Δτ*_rise,*i*_ and *Δτ*_fall,*i*_ were determined in the same way. The characteristics varying on different scales, the differences were weighted and summed to produce a single cost function
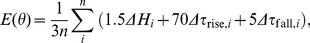
(42)where *n*  =  26 is the number of uptakes. Third, a solution was finally accepted only if it was in qualitative accordance with other available experimental facts (transient kinetics of DAG- and IP_3_-gated currents, sustained Cl^−^ and K^+^ currents, intracellular Ca^2+^ concentration below 200 µM). In the present work, no global exploration of the parameter space by trial-and-error was needed because all parameter values were kept the same as in [18], except for 7 parameters. The solutions were locally optimized utilizing the Matlab unconstrained minimizer *fminsearch* based on the Nelder-Mead simplex method. The algorithm converged on the set of estimated parameters *θ*_0_ shown in Table 3.

## Supporting Information

Figure S1 **Electrical parameters influencing the height of RP at soma and SP.** (A) Transepithelial potential *E*_a_ at auxiliary cells. (B) Leak battery at soma *E*_ls_. (C, D) Leak conductance *G*_ls_ at the inner dendrite and soma. (E) Conductance *G*_a_ at auxiliary cells. (F) Capacitance *C*_a_ at auxiliary cells. Effects shown at low (0.1 nS), intermediate (1 nS) and high (10 nS) pheromone-dependent conductance *G*_p_. The vertical dotted lines indicate the biologically realistic parameter values given in Tables 1 and 2.
(DOC)

Figure S2 **Electrical parameters influencing the rising and falling times of RP and SP.** Rising times (left column) and falling times (right column) of RP at soma (blue lines) and SP (red lines). (A, B) Conductance *G*_a_ at auxiliary cells. (C, D) Capacitance *C*_a_ at auxiliary cells. (E, F) Capacitance *C*_d_ at outer dendrite. Effect shown at low (0.1 nS), intermediate (1 nS) and high (10 nS) pheromone-dependent conductance *G*_p_. *G*_ls_ and *C*_s_ have weak influence on the rising and falling time, *E*_a_ and *E*_ls_ have no influence on the transient process. The vertical dotted lines indicate the biologically realistic parameter values given in Tables 1 and 2.
(DOC)

Figure S3 **Electrical parameters influencing the amplification factor of RP at soma.** (A) Equilibrium potential of auxiliary cells *E*_a_. (B) Equilibrium potential of leak current of soma *E*_ls_. (C) Leak conductance of soma *G*_ls_. (D) Leak conductance of auxiliary cells *G*_a_. (E) Capacitance of soma *C*_s_. (F) Capacitance of auxiliary cell *C*_a_. All parameters are influential except *C*_d_ (not shown).
(DOC)

Figure S4 **Electrical parameters influencing the relative amplification factor of RP at soma as a function pheromone-dependent conductance** ***G*** **_p_.** (A) Leak conductance at soma *G*_ls_. (B) Capacitance of auxiliary cell membranes *C*_a_. For all other electrical parameters the curves obtained are practically superimposed and correspond to the curves for *G*_ls_  =  1.5 nS and *C*_a_  =  3.5 pF. The vertical dotted lines indicate the range of *G*_p_ from 6.4×10^−2^ to 4 nS corresponding to the pheromone uptake rang from 10^−4.75^ to 10^1.5^ µM/s.
(DOC)
