# Supplementary material for: Dynamical Modeling of the Moth Pheromone-Sensitive Olfactory Receptor Neuron within Its Sensillar Environment
Source: PLoS One. 2011 Mar 2;6(3):e17422. doi: 10.1371/journal.pone.0017422 (PMC3047557; doi:10.1371/journal.pone.0017422)
Supplement: Figure S1 — Electrical parameters influencing the height of RP at soma and SP. (A) Transepithelial potential E a at auxiliary cells. (B) Leak battery at soma E ls. (C, D) Leak conductance G ls at the inner dendrite and soma. (E) Conductance G a at auxiliary cells. (F) Capacitance C a at auxiliary cells. Effects shown at low (0.1 nS), intermediate (1 nS) and high (10 nS) pheromone-dependent conductance G p. The vertical dotted lines indicate the biologically realistic parameter values given in Tables 1 and 2. (DOC) [file pone.0017422.s001.doc]

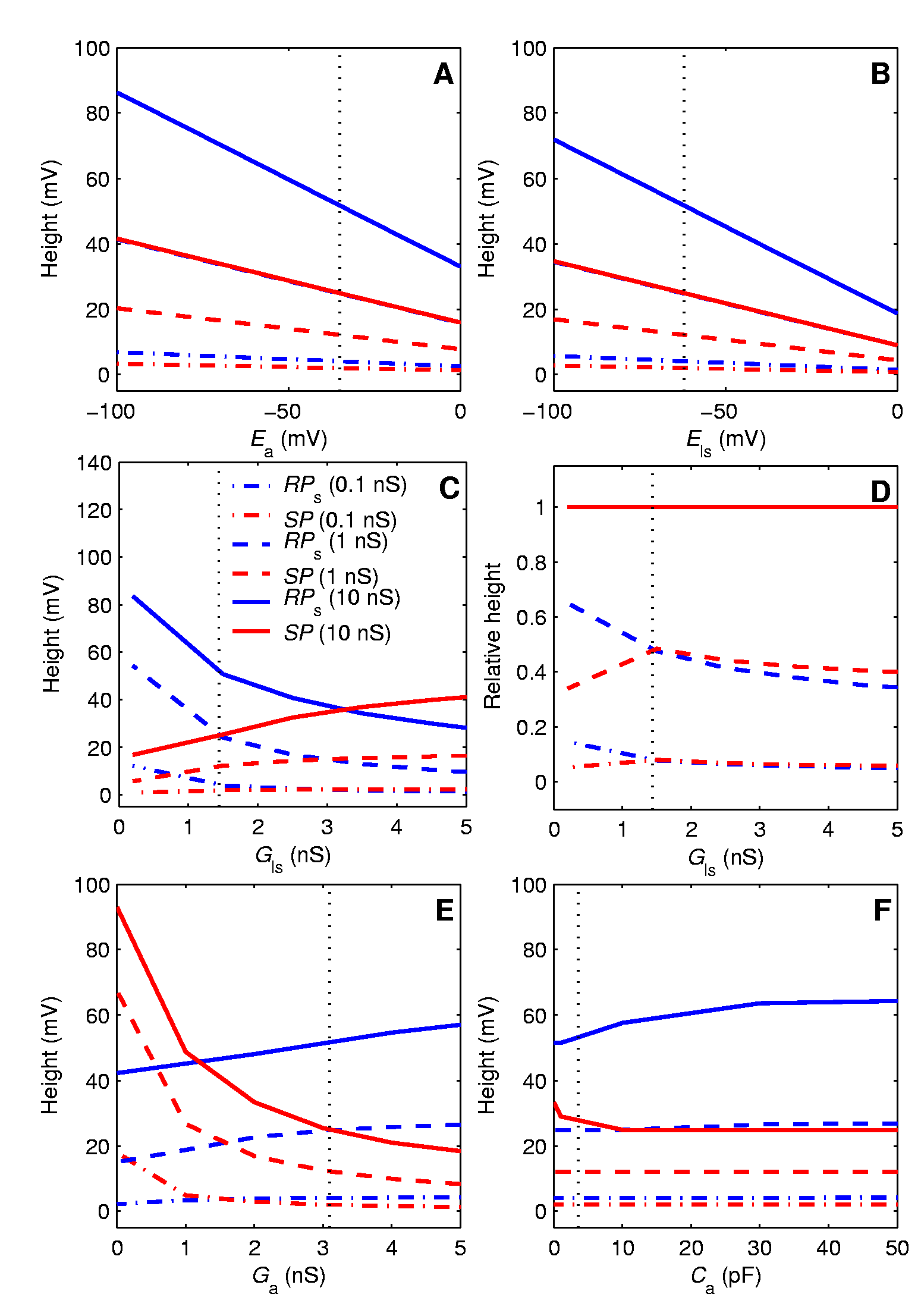


**Figure S1. Electrical parameters influencing the height of RP at soma and SP**. (A) Transepithelial potential *E*_a_ at auxiliary cells. (B) Leak battery at soma *E*_ls_. (C, D) Leak conductance *G*_ls_ at the inner dendrite and soma. (E) Conductance *G*_a_ at auxiliary cells. (F) Capacitance *C*_a_ at auxiliary cells. Effects shown at low (0.1 nS), intermediate (1 nS) and high (10 nS) pheromone-dependent conductance *G*_p_. The vertical dotted lines indicate the biologically realistic parameter values given in Tables 1 and 2.
