# Supplementary material for: Dynamical Modeling of the Moth Pheromone-Sensitive Olfactory Receptor Neuron within Its Sensillar Environment
Source: PLoS One. 2011 Mar 2;6(3):e17422. doi: 10.1371/journal.pone.0017422 (PMC3047557; doi:10.1371/journal.pone.0017422)
Supplement: Figure S2 — Electrical parameters influencing the rising and falling times of RP and SP. Rising times (left column) and falling times (right column) of RP at soma (blue lines) and SP (red lines). (A, B) Conductance G a at auxiliary cells. (C, D) Capacitance C a at auxiliary cells. (E, F) Capacitance C d at outer dendrite. Effect shown at low (0.1 nS), intermediate (1 nS) and high (10 nS) pheromone-dependent conductance G p. G ls and C s have weak influence on the rising and falling time, E a and E ls have no influence on the transient process. The vertical dotted lines indicate the biologically realistic parameter values given in Tables 1 and 2. (DOC) [file pone.0017422.s002.doc]

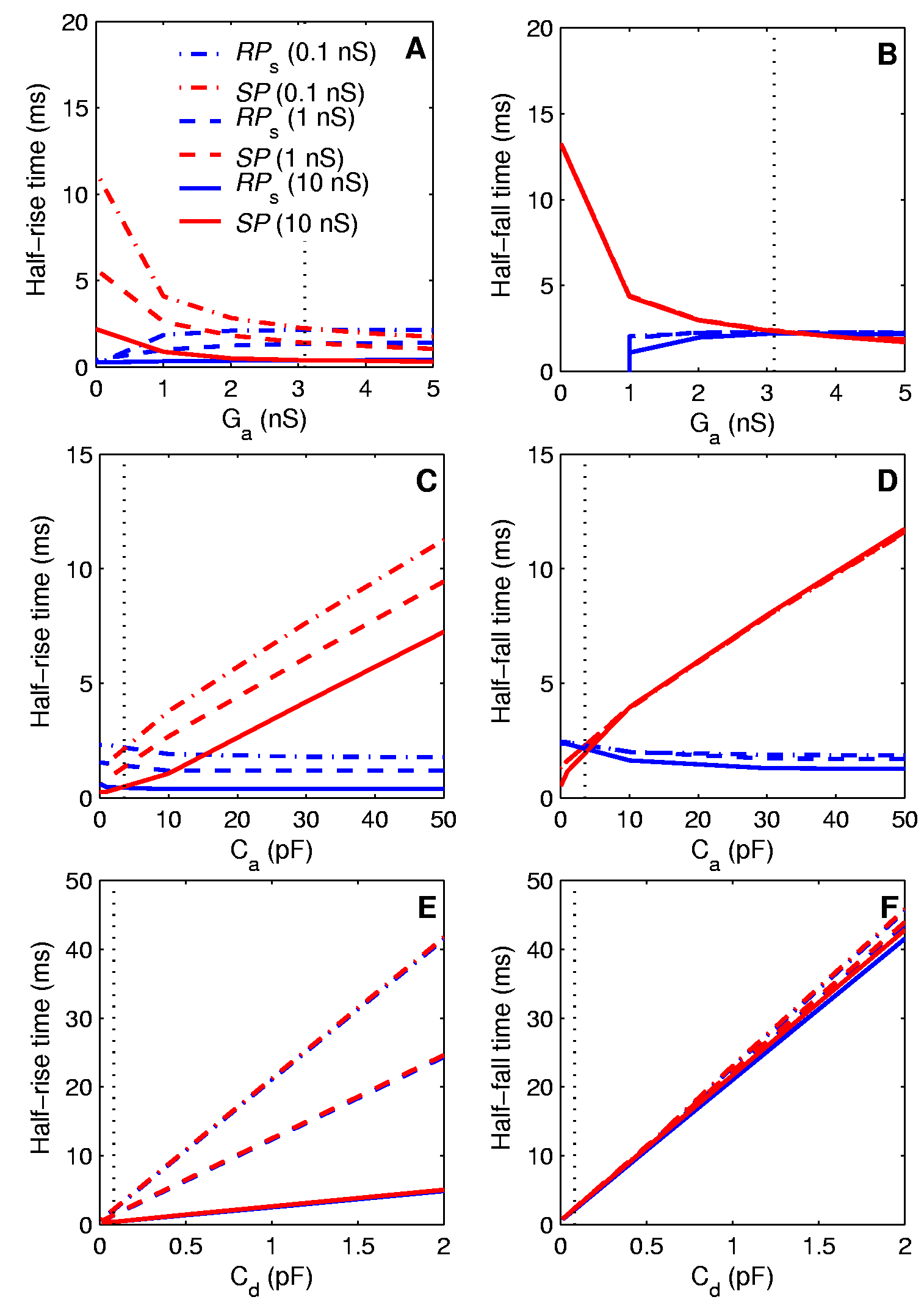


**Figure S2. Electrical parameters influencing the rising and falling times of RP and SP.** Rising times (left column) and falling times (right column) of RP at soma (blue lines) and SP (red lines). (A, B) Conductance *G*_a_ at auxiliary cells. (C, D) Capacitance *C*_a_ at auxiliary cells. (E, F) Capacitance *C*_d_ at outer dendrite. Effect shown at low (0.1 nS), intermediate (1 nS) and high (10 nS) pheromone-dependent conductance *G*_p_. *G*_ls_ and *C*_s_ have weak influence on the rising and falling time, *E*_a_ and *E*_ls_ have no influence on the transient process. The vertical dotted lines indicate the biologically realistic parameter values given in Tables 1 and 2.
