# Supplementary material for: Dynamical Modeling of the Moth Pheromone-Sensitive Olfactory Receptor Neuron within Its Sensillar Environment
Source: PLoS One. 2011 Mar 2;6(3):e17422. doi: 10.1371/journal.pone.0017422 (PMC3047557; doi:10.1371/journal.pone.0017422)
Supplement: Figure S3 — Electrical parameters influencing the amplification factor of RP at soma. (A) Equilibrium potential of auxiliary cells E a. (B) Equilibrium potential of leak current of soma E ls. (C) Leak conductance of soma G ls. (D) Leak conductance of auxiliary cells G a. (E) Capacitance of soma C s. (F) Capacitance of auxiliary cell C a. All parameters are influential except C d (not shown). (DOC) [file pone.0017422.s003.doc]

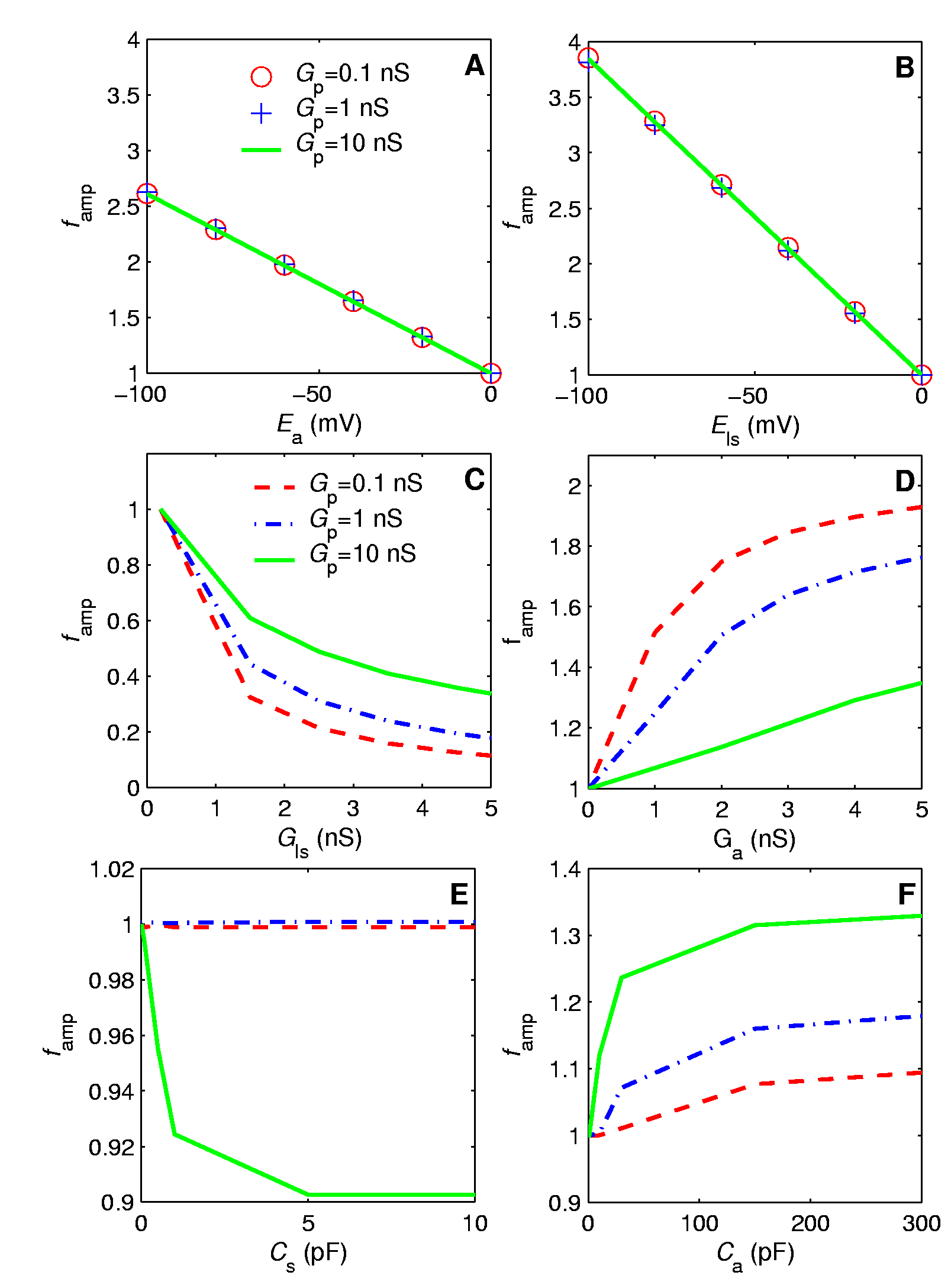


**Figure S3. Electrical parameters influencing the amplification factor of RP at soma**. (A) Equilibrium potential of auxiliary cells *E*_a_. (B) Equilibrium potential of leak current of soma *E*_ls_. (C) Leak conductance of soma *G*_ls_. (D) Leak conductance of auxiliary cells *G*_a_. (E) Capacitance of soma *C*_s_. (F) Capacitance of auxiliary cell *C*_a_. All parameters are influential except *C*_d_ (not shown).
