# Supplementary material for: Dynamical Modeling of the Moth Pheromone-Sensitive Olfactory Receptor Neuron within Its Sensillar Environment
Source: PLoS One. 2011 Mar 2;6(3):e17422. doi: 10.1371/journal.pone.0017422 (PMC3047557; doi:10.1371/journal.pone.0017422)
Supplement: Figure S4 — Electrical parameters influencing the relative amplification factor of RP at soma as a function pheromone-dependent conductance G p. (A) Leak conductance at soma G ls. (B) Capacitance of auxiliary cell membranes C a. For all other electrical parameters the curves obtained are practically superimposed and correspond to the curves for G ls = 1.5 nS and C a = 3.5 pF. The vertical dotted lines indicate the range of G p from 6.4×10^−2^ to 4 nS corresponding to the pheromone uptake rang from 10−4.75 to 101.5 µM/s. (DOC) [file pone.0017422.s004.doc]

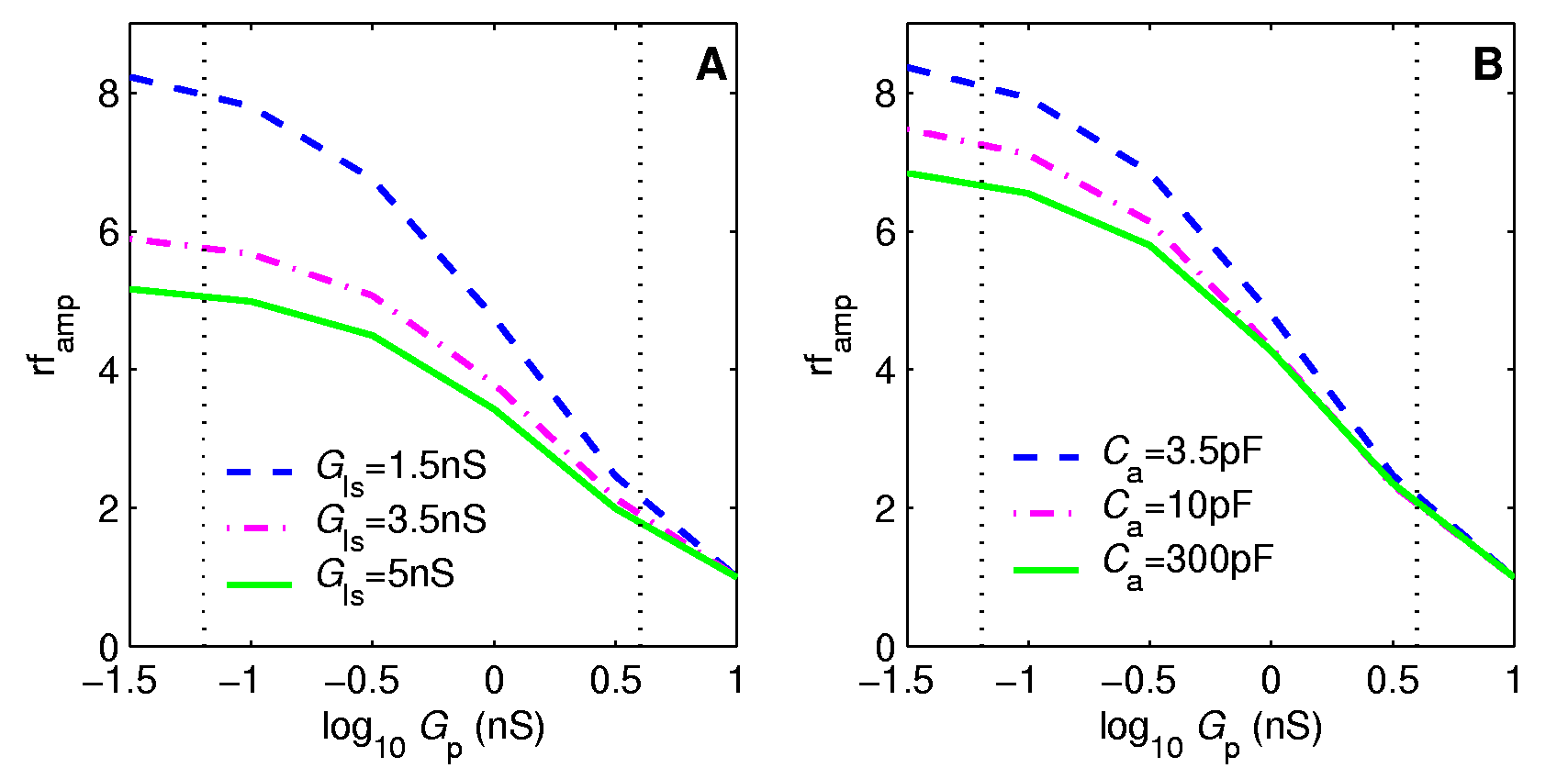


**Figure S4. Electrical parameters influencing the relative amplification factor of RP at soma as a function pheromone-dependent conductance *G*_p_**. (A) Leak conductance at soma *G*_ls_. (B) Capacitance of auxiliary cell membranes *C*_a_. For all other electrical parameters the curves obtained are practically superimposed and correspond to the curves for *G*_ls_ = 1.5 nS and *C*_a_ = 3.5 pF. The vertical dotted lines indicate the range of *G*_p_ from 6.4 × 10^−2^ to 4 nS corresponding to the pheromone uptake rang from 10^−4.75^ to 10^1.5^ µM/s.
